# High-Throughput Screening of Molecule/Polymer Photocatalysts for the Hydrogen Evolution Reaction

**DOI:** 10.1021/acscatal.5c01785

**Published:** 2025-04-10

**Authors:** Lei Shi, Alessandro Troisi

**Affiliations:** Department of Chemistry, University of Liverpool, Liverpool L69 7ZD, U.K.

**Keywords:** high-throughput virtual screening, microkinetic modeling, hydrogen evolution reaction, organic photocatalysis, density functional theory, time-dependent density functional theory

## Abstract

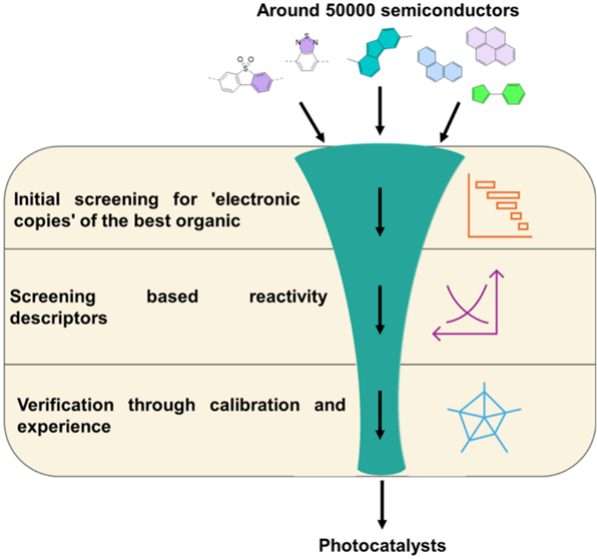

Although there has been progress in designing organic photocatalysts, identifying and designing structurally distinct polymeric or molecular photocatalysts with high performance is still challenging. Using the properties of a set of well-known polymer photocatalysts, we performed a virtual screening of a large data set of around 50 000 organic semiconductors. In the initial stage, we looked for candidates with electronic properties similar to those of the best-performing photocatalysts. Next, we screened the data set using reactivity descriptors based on mechanisms derived from quantum chemical calculations for selected cases. We identified 33 candidates with high potential as photocatalysts for the hydrogen evolution reaction.

## 1 Introduction

Linear polymer/molecular photocatalysts are able to utilize visible light in the development of artificial photosynthesis^1−3^ and green organic synthesis.^4,5^ More specifically, these organic materials show potential in the photocatalyzed hydrogen evolution reaction (HER) with low-cost but high performance, rivaling inorganic photocatalysts.^6−8^ However, organic photocatalysts still cannot be used in practical applications under natural sunlight because of their low and unstable long-term activity.^7,9^ The systematic identification of novel photocatalysts, beyond simple modification of the existing ones, often proceeds through a combination of several approaches. Chemical automation has enabled the testing of hundreds of compounds for their photocatalytic HER,^10,11^ and these data are intrinsically homogeneous and very well suited for machine learning (ML) predictions of new compounds.^6,12,13^ On the other hand, experimental data sets remain limited by their cost, and ML predictions become less accurate (generalizable) for hypothetical compounds far beyond the feature space occupied by the training set.^14,15^ For this reason, it is desirable to use computational chemistry methods to explore a much broader chemical space to develop physical models of reactivity that can be applied to very diverse chemical species, ideally unrelated to those under current investigation.

The high-throughput virtual screening (HTVS) approach is an effective complementary approach to discover new interesting lead compounds with novel chemistry and has been successfully applied in thermally activated delayed fluorescence (TADF),^16^ organic light-emitting diode (OLED)^17^ materials, and several kinds of inorganic photocatalysts or metal–organic frameworks.^18−23^ Current HTVS screening studies^6,12,24,25^ of the HER on organic photocatalysts are based on the computation of excitation and charge transfer properties relating to primary reactions, including light absorption and charge transport, such as exciton electron affinity, exciton binding energy, optical gap, and singlet–triplet energy gap. However, these studies do not consider descriptors pertinent to critical secondary reactions, including H^+^ adsorption and H–H coupling. High-throughput experimentation generally requires hundreds of molecules.^11,12^ Therefore, it is necessary to explore organic photocatalysts in ample chemical space by combining the properties related to the primary reaction and a comprehensive understanding of the secondary reaction of the HER.

In this work, we first analyzed the mechanism of the HER for the 12 best-known catalysts with C and N active sites in the presence of a sacrificial agent (SA) triethylamine^26^ (TEA), with unintended small Pd residue (generally below 1.1 wt %). We neglected the effect of cocatalyst,^27−29^ like metals, and focused only on the polymer performance. We explored the reaction and activation energy of the elementary steps and found a Bell–Evans–Polanyi (BEP) relation between them. Microkinetic models were then built to identify the potential descriptors. By combining the analysis of the general properties of the 8 best-performing photocatalysts and our microkinetic results, we found 33 novel candidates across four different types of reaction sites that can be used for future photocatalyst design.

## 2 Methods

**Figure 1:**
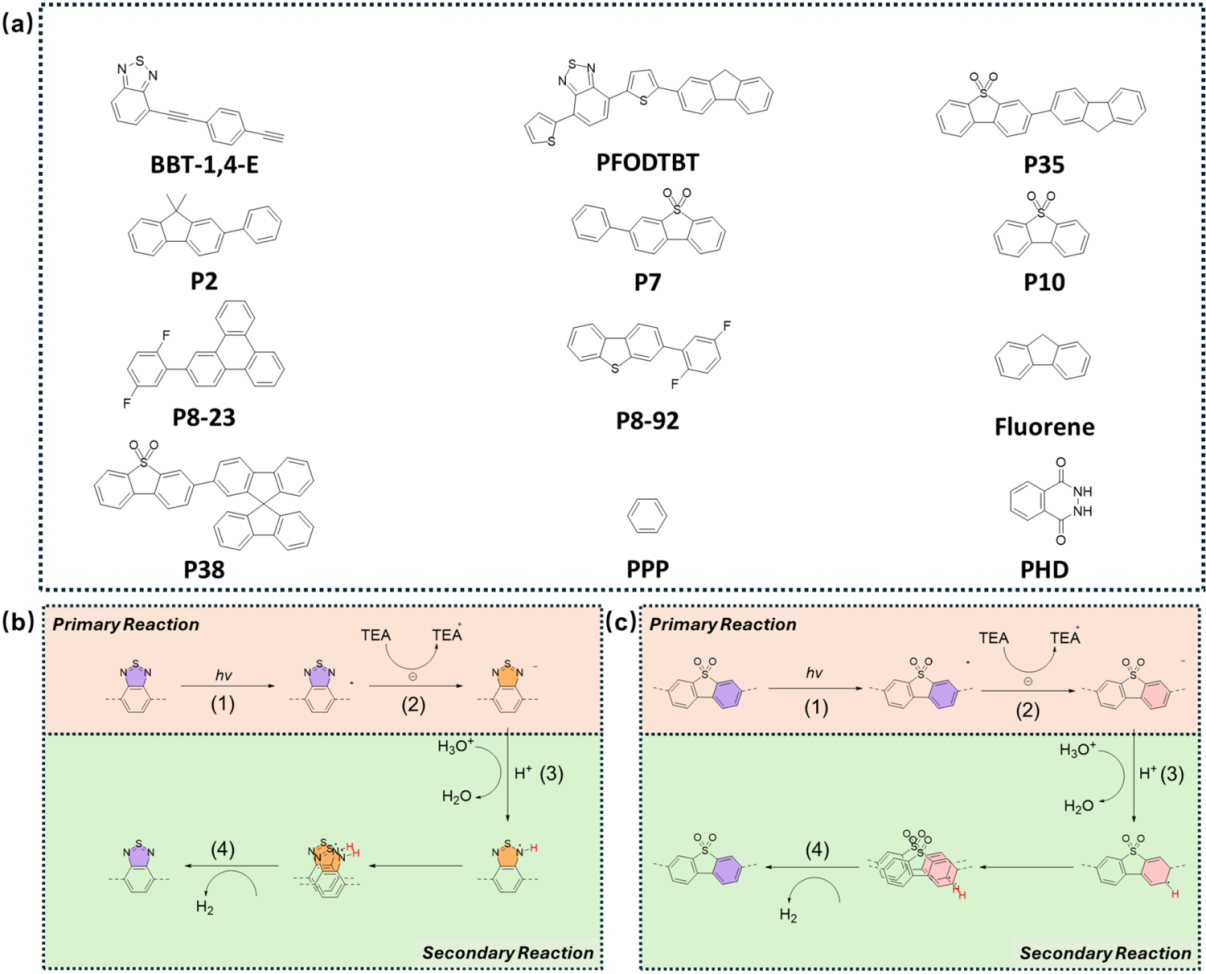
(a) Structures of the repeat units. Schematic diagrams of the (b) reaction mechanism of N-site molecules and (c) reaction mechanism of C-site molecules.

To identify the general mechanism, we selected 12 known polymer photocatalysts possessing two different active sites (repeat units are shown in Figure 1, with the labels used in the main manuscript and Table S1, with some of their full names) with high activity in the HER reaction in previous studies. These polymers with C active sites include PPP from the group of Shozo Yanagida,^30^ P2, P7, P10, P35, P38, P8–23, and P8–92 from the group of Cooper.^6,31^ Others with N active sites include PFODTBT from the group of Araujo and Tian^32^ and BBT-1,4-E from the group of Chen and Zhu.^33^ They are considered good representatives of the class of polymeric photocatalysts. Two typical organic photocatalyst structures,^34,35^ including PHD and fluorene, were also selected here to expand our data set. The polymer monomer, an oligomer containing one unit, and dimers, an oligomer containing two units, were used to model molecule/linear polymer catalysts. We verified that these models were sufficient for the descriptors extracted from them. Density functional theory (DFT), unrestricted DFT, and time-dependent DFT (TD-DFT) calculations with the 6-31G(d,p) basis set^36^ were carried out using Gaussian 16 to obtain the energies of the polymers in the S_0_ state, radical, and S_1_ or T_1_ states, respectively. B3LYP with dispersion correction using Becke–Johnson damping (D3BJ)^37^ corrects for missing London dispersion and forces at shorter distances, considering the good balance between computational cost and accuracy in the thermodynamics of organic reactions.^38−40^ The free energy of the relevant intermediates was computed from the internal energy of Gaussian 16 (revision A.03)^41^ using a harmonic approximation at 298.15 K. The implicit continuum solvation (IEFPCM) model^42,43^ was utilized to describe the solvent effect of water on Gibbs free energy in HER mechanism studies because of its low cost and high accuracy in estimating solvation-free energy.^44,45^ The enthalpy and entropy correction for the H_2_ gas was evaluated by using the Shomate equation.^46^

### 2.1 Database

To identify potential novel photocatalysts, we screened a data set of 48,182 organic semiconductors (computed highest occupied molecular orbital–lowest unoccupied molecular orbital (HOMO–LUMO) gap below 4 eV) presented in ref (47) and derived from the Cambridge Structural Database (CSD). This data set is reliable and convenient for searching real organic photocatalysts because these molecules can produce stable solid crystals with documented synthetic pathways. Besides, the critical electronic properties include the energies of the first three singlet excited states *E*(S_1_), *E*(S_2_), and *E*(S_3_), and the corresponding oscillator strengths *f*(S_1_), *f*(S_2_), and *f*(S_3_). The sum of oscillator strengths provides an acceptable indication of the ability of these compounds to absorb radiation. These properties were calculated using TD-DFT with the M06-2X functional and def2-SVP basis set with errors within 0.05 eV (root mean square error (RMSE))^47,48^ between experiments and calculations.

## 3 Results and Discussion

### 3.1 Mechanism and Rate-Determining Step (RDS)

The initial stage of this screening process is the identification of the mechanisms and the rate-determining step. We categorized the elementary steps of the HER into two groups (see Figure 1a–c), including primary reactions, such as photoexcitation (step 1) and electron transfer (step 2) processes, and secondary reactions, such as hydrogen ion adsorption and their coupling (steps 3 and 4). The primary reactions are not the focus of this study, but a brief outline is given. The free energies of step 1 and step 2 can be calculated as follows:
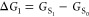
1
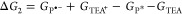
2where *G*_P*_ and *G*_P_ are the free energies of the polymer in the S_1_ and S_0_ states, respectively, and *G*_P^•–^_ and *G*_TEA^+^_/*G*_TEA_ are the free energies of the polymer with an extra electron and TEA^+^/TEA, respectively. Δ*G*_2_ is typically below 0 eV (see Table S2).

The electron transfer rate (*k*_et_) between the catalyst and TEA is assumed to be rapid. This is a reasonable assumption for organic material catalysts without metal cocatalysts based on the reality that the efficiencies of many photoredox-catalyzed reactions exhibit quantum yields well below 1, which is maximum if only the photoinduced electron transfer cycles are operating.^49^ For example, T-BODIPY (thiophene at the meso-position) and T-ADA (based on benzodithiophene as the donor and BODIPY as the acceptor) were found to have much higher charge-separation rates (100–200 ps) than their charge recombination rates (3–8 ns).^50^ The time scale of intramolecular electron transfer for betaines is within 5.5 ps.^51^ The phenazine-based photoactive^52^ can achieve a high bimolecular dissociative photoinduced electron transfer rate (∼10^9^ s^–1^).^53^

The proposed secondary reaction mechanism of the HER on polymers in our study (see Figure 1b,c) starts with the adsorption of H^+^ (step 3, analogous to the Volmer step^33,54−56^). The two adsorbed H atoms can combine to generate H_2_ (step 4, analogous to the Tafel step^33,55−58^). The H^+^ from the solvent can couple with adsorbed H on polymers to generate H_2_ (step 5, analogous to the Heyrovsky step,^33,54−56^ see eq 11). The free energies of steps 3–5 can be calculated as follows:
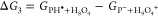
3
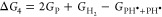
4
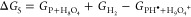
5where *G*_H_2__ and *G*_PH^•^_ are the free energies of the hydrogen gas and polymer anions with an H^+^ adsorbate, respectively. Previous literature reveals that the [H_3_O(H_2_O)_3_]^+^ cluster is stable and suitable for studying hydration reactions^59^ and proton transfer reactions.^60^ Therefore, to model H^+^ transfer between the polymer/water interface (see Tables S3 and S4 and Figure S1), we used a polymer anion with an H_9_O_4_^+^ cluster (P^•–^ + H_9_O_4_^+^) for the initial state and a polymer radical with an H_8_O_4_ cluster (PH^•^ + H_8_O_4_) for the final state. This can be regarded as a reaction initiated by an infinite distance between the reactants. We refer to these quantities as Δ*G*_3-ref_ and Δ*G*_4-ref_. The free energy can be expressed as
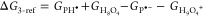
6
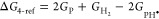
7
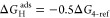
8
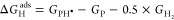
9It is useful to define Δ*G*_3-ref_ and Δ*G*_H_^ads^ as descriptors because they can be straightforwardly calculated by using the energy of water clusters, polymers, and hydrogen gas separately instead of the complex clusters of initial states. Δ*G*_H_^ads^ can be considered as the adsorption of the H atom on a polymer, which is equal to −0.5Δ*G*_4-ref_. Δ*G*_3-ref_ can be considered as the reaction energy of the H^+^ and PH^•^ approach from an infinite distance away instead of in a neighboring site (Δ*G*_3_). Expressions similar to those proposed for Δ*G*_H_^ads^ and Δ*G*_3-ref_ were used as descriptors in the electrocatalysis of the HER on transition metals^61^ and photocatalysis of the HER on Pt/TiO_2_,^55^ respectively.

We further calculated the reaction energies (Δ*G*_3_ and Δ*G*_3-ref_) and activation energy barrier of H^+^ adsorption on the P^–^ reaction (Δ*G*_3_^a^) (see Table S4), and the relevant structures and energy are shown in Figure S1 and Table S3. We found that the adsorption of H^+^ was exergonic for all polymer anions. The activation energy barrier varies from 0.03 to 0.49 eV on the carbon sites (see Table S4), while there is a zero activation energy barrier on the N site of the polymer (BBT-1,4-E and PFODTBT). This indicates that step 3 is more likely to be a spontaneous reaction on most polymers with N sites (2,1,3-benzothiadiazole group) and some polymers with C sites (like P2, P8–23, and PPP dimer). The adsorption of H^+^ on N sites was much easier. We then studied the possible length effect of the polymers in step 1 by extending our calculations to longer chains, including the PPP dimer, P10-dimer, P38-dimer, and PPP4. The free reactions and activation energies of several dimer anions were predicted to be higher than those of their monomers, indicating that the increased chain length may inhibit H^+^ adsorption on polymer anions. Although we did not extend our calculations to longer chains due to the high cost of calculating the transition states, the current results can describe the possible effects of length. Furthermore, in the next section, we found that the dimers and monomers follow a general relationship that can be used to explore the potential impact of the length variation of compounds on adsorption energy. Our models were thus sufficiently large to reach a general conclusion.

H_2_ may be generated through Heyrovsky or Volmer paths for different catalysts.^55,56,62−64^ Although the Tafel step has been explored on several organic catalysts in previous theoretical studies,^32,33,58^ to the best of our knowledge, the major path of H_2_ generation has not been studied systematically on polymer photocatalysts. In the next section, we explore both paths and discuss the most favored path for H_2_ generation.

#### 3.1.1 Tafel-2P Path

In the 2P path, H^+^ is first adsorbed onto two different polymer anions to form two radicals (2PH^•^). The two adsorbed H atoms on two adjacent radicals combine to form H_2_, and the elementary reaction is as follows:
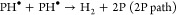
10The reaction free energy and activation energy barrier were calculated (see Table 1) using radicals (2PH^•^) with a triplet electronic state as the initial state. The relative structures are shown in Figure S2. Besides, the activation energy barrier values on molecules with the C active site range from 0.29 to 0.58 eV, which is significantly smaller than that on the N site (BBT-1,4-E: 1.51 eV and PFODTBT: 1.24 eV).

**Table 1:** Free Reaction Energy and Activation Energy Barrier for Heyrovsky and Tafel Steps (eV)^a^

polymers	Δ*G*_elec_	Δ*G*_5_	Δ*G*_5_^a^	Δ*G*_4-2P_	Δ*G*_4-2P_^a^	Δ*G*_4-1P_	Δ*G*_4-1P_^a^
BBT-1,4-E	1.96	–1.60	1.12	–1.12	1.51	–0.41	2.97
PFODTBT	1.31	–0.95	1.44	–1.28	1.24	–0.52	2.87
PPP4	2.17	–2.55	0.26	–2.52	0.36	–1.86	1.76
PPP dimer	2.36	–2.73	0.17	–2.56	0.48	–1.93	1.69
PPP-mono				–2.84	0.29		
P2	2.16	–2.49	0.49	–2.46	0.33	–1.84	0.98
P7	1.28	–1.41	0.87	–2.32	0.37	–0.81	1.07
P8–23	1.95	–2.01	0.51	–2.12	0.58	–0.97	0.97
P8–92	1.97	–2.24	0.64	–2.36	0.42	–0.92	1.23
P10-dimer	1.26	–1.51	0.79	–2.3	0.39	–2.50	0.96
P10	1.32	–1.64	0.82	–2.34	0.38	–1.74	1.25
P35						–0.61	1.06
P38	1.59	–1.80	0.63	–2.24	0.44	–0.27	1
P38-dimer	1.66	–1.91	0.53	–2.3	0.44	–1.66	1.02
fluorene	2.58	–2.80	0	–2.84	0.37	–1.47	1.25
PHD	1.40	–2.08	0.72	–2.5	0.37		

a The transition state or initial state was not located for these species.

To compute the activation energy, we first consider the stability of the initial state where two paired radicals (2PH^•^) may be in the singlet state or in the triplet state (the free energy reaction pathways are reported in a graphical form in Figures S2–S5). In the singlet state, the free energy of the 2PH^•^ conformation for P10, PPP, P2, P38, P35, P8–92, P8–23, and P38 in the singlet state was predicted to be smaller than those in the triplet state (see Figure S3). These molecules, with a more stable singlet electronic state, are more likely to form a single bond between the two monomers (see Figure S5), leading to a PH–PH dimer. This chain-termination reaction may affect the concentration of PH^•^ as a side reaction. In contrast, others, including P7, P2, PFODTBT, and BBT-1,4-E, have a more stable triplet electronic state, which is the initial state of the Tafel-2P path. One can expect better HER activity for these molecules due to their less favorable chain-termination reaction. Considering the possible length growth effect, we next calculated the free energy of 2PH^•^ of dimer structures for PPP, P2, P38, P10, and P7 in the singlet and triplet states. The triplet state was found to be more stable with increasing chain length, indicating that this side reaction is less likely to occur with a longer chain. This also suggests that 2PH^•^ with a stable singlet state may also form a longer length to participate in the 2PH^•^ coupling step in the triplet state. Thus, the activation energy is calculated by using the triplet-state energy as the initial state (see Figure S4).

Another pathway of H_2_ generation on organic catalysts, analogous to the Heyrovsky step, can be expressed as follows:
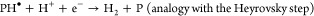
11where PH^•^ couples with H^+^ from water and is accompanied by an electron transfer step. We first examined the free reaction energy of the electron transfer step from TEA to polymer Δ*G*_elec_, which can be calculated as
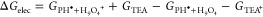
12Table S5 shows the relative structures of the intermediate and transition states. We found that the electron transfer is an endergonic process with a free energy of >1.28 eV (see Table 1) for our data set. This suggests that the electron transfer step is difficult for polymers. Upon electron transfer, hydrogen transfer from water to adsorbed H generates H_2_ with an activation energy barrier of 0.17–0.82 eV on the C site and activation energy barriers of 1.24 and 1.51 eV on the N site (see Table 1). Therefore, the Heyrovsky step is less favorable than the 2P path.

#### 3.1.2 1P Path

Another path for H–H coupling is that the two H^+^ can adsorb at adjacent sites of the same polymer to form a PH_2_ intermediate. The H_2_ is generated after two H are coupled, and the reaction can be expressed as follows:
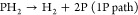
13The free energy (Δ*G*_4-1P_), activation energy barrier (Δ*G*_4-1P_^a^), and structures of each state are presented in Tables 1 and S6. For the C active site molecules, in the initial state, two H^+^ are adsorbed on the most favored site and the neighboring site, which is on a neighboring benzene molecule, separately. For BBT-1,4-E and PFODTBT with N sites, two H^+^ are adsorbed on the two N sites. Two H atoms are coupled to generate H_2_, and the distance of the H–H bond in the transition state is equal to 1.06–1.13 Å. The 1P path is less favored because its activation energy barrier is around twice that of the 2P path (see Table 1). This is consistent with what has been found in previous literature that the activation energy barrier of the 2P path is much larger than that of the 1P path on benzothiadiazole, BBT-1,4-E, and poly(*p*-phenylene).^32,33,58^ We also extended our calculation of the activation energy barrier for the P10-dimer, P38-dimer, and PPP4 and found that the chain length had little influence on the activation barrier. Thus, we considered the Tafel-2P path in the H_2_ generation mechanism and ignored the Tafel-1P path.

### 3.2 Microkinetic Model: Determining Convenient Reactivity-Based Descriptors for Screening

The turnover frequency (TOF) is a widely adopted concept to evaluate the intrinsic activity of organic catalysts.^65,66^ We developed a mean-field microkinetic model to evaluate the turnover frequency (TOF) defined as the rate of production formed per unit time per number of active sites^67^ of the HER under the assumption of a rate-demining step (details in Supporting Information (SI)).^55,68^ A similar approach to TOF-based calculations was used in previous work to evaluate nanostructured carbon materials.^69^ This model allows us to express the rate of the overall process in terms of easily computable quantities Δ*G*_3-ref_ and Δ*G*_H_^ads^ by combining microkinetic and linear relations. The elementary steps (steps 3 and 4, see Figure 1) included in the model are as follows:
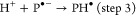
14
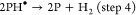
15

The rate of the elementary steps 3 and 4 is thus proportional to the concentration of each species, and the reaction rate equation of the elementary steps 3 and 4 is expressed as
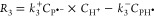
16
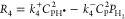
17where *C*_P^•–^_ and *C*_P_ are the concentrations of the polymer anions and polymers, respectively, *C*_H^+^_ is the concentration of the hydrogen cations, *C*_PH^•^_ is the concentration of PH^•^ in the liquid, and *P*_H_2__ is the pressure of hydrogen gas.

**Figure 2:**
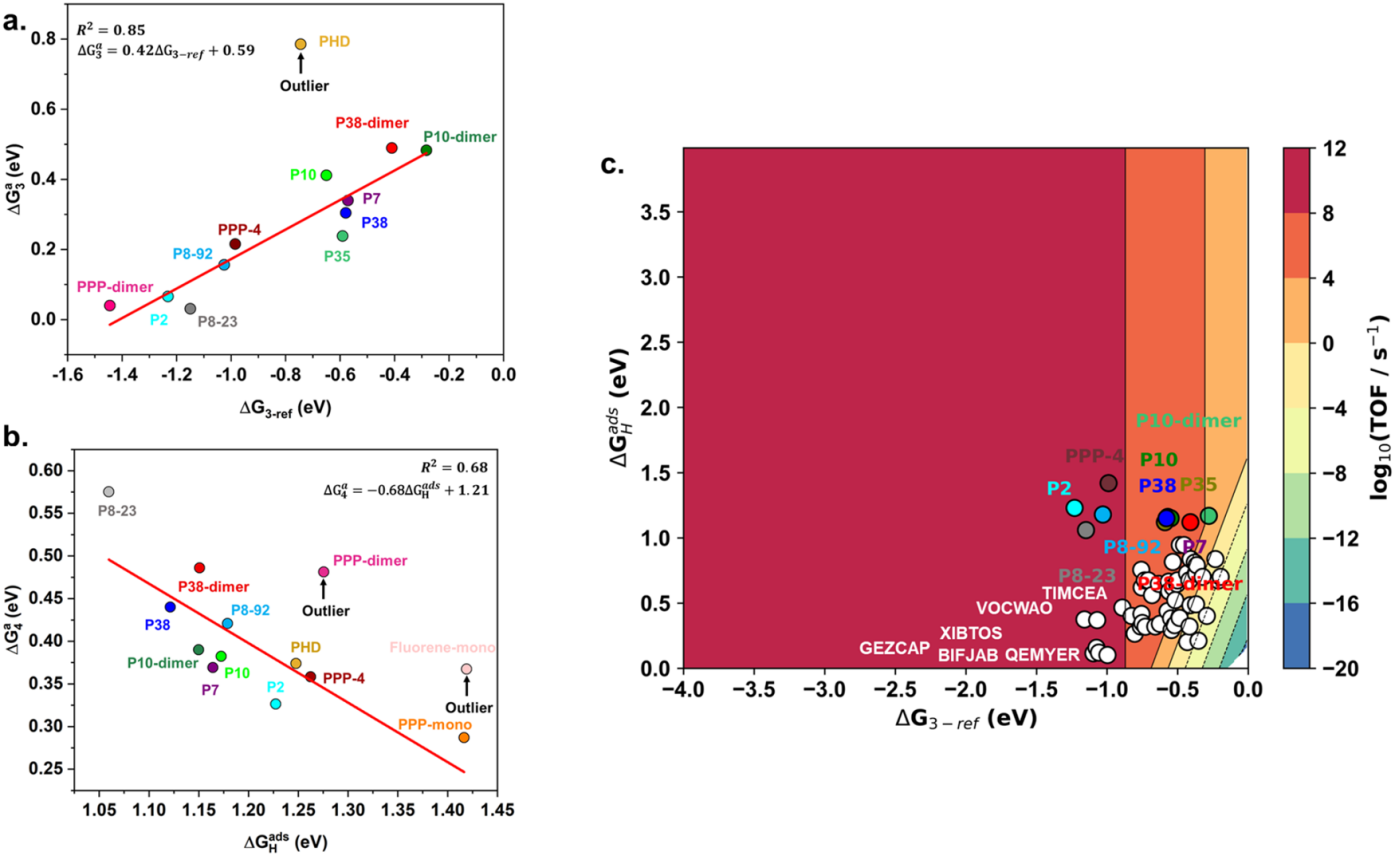
(a) Scaling relations of the descriptor of reaction 3 (Δ*G*_3-ref_) vs the activation energy barrier (Δ*G*_3_^a^), excluding PPP-mono and fluorene (transition state has not been located). (b) Scaling relations of the descriptor of reaction 4 (Δ*G*_H_^ads^) vs the activation energy barrier (Δ*G*_4_^a^), excluding PHD and P35 (the transition state was not located). (c) TOF of the calculated C site molecules at a concentration of P^•–^ at 0.1 mol L^–1^.

The total rate can be evaluated using the minimum values of *R*_3_ and *R*_4_:
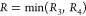
18We focused on the relationship between the reaction energy and activation energy, the Bell–Evans–Polanyi (BEP) relation,^70^ for the H^+^ adsorption step to further simplify the computational cost in transition states. We plotted Δ*G*_3-ref_ vs Δ*G*_3_ (see Figure S6a) and Δ*G*_3-ref_ vs Δ*G*_3_^a^ (see Figure 2a), where Δ*G*_3-ref_ refers to the reaction energy when one reactant is at an infinite distance from the other, and Δ*G*_H_^ads^ refers to the adsorption energy of an H atom on an active site (as detailed in the Methods section). By using Δ*G*_3-ref_, the energies of the solvent and polymer can be calculated separately, allowing us to estimate the free energy. This approach simplifies the process, as it only requires the calculation of *G*_PH^•^_, *G*_P_, *G*_H_2__, *G*_H_8_O_4__, *G*_P^•–^_ and *G*_H_9_O_4_^+^_ instead of modeling the complex solvent–polymer interface for both the initial and final states. We observed an excellent linear relationship between Δ*G*_3-ref_ and Δ*G*_3_ (see Figure S6a) across different types of polymers on C sites, excluding the PHD that does not follow the BEP trend well. The free reaction energy of protonation increases with an increase in the reference free energy (Δ*G*_3-ref_). It should be noted that the N sites are excluded here as they have a zero-energy barrier.

A similar analysis was performed for Tafel-2P paths, as shown in Figure 2b. It can be seen that the free reaction energy (Δ*G*_4_) of H–H coupling (Tafel-2P path) (see Figure S6b) and activation energy barrier (Δ*G*_4_^a^) (see Figure 2b) for molecules with only C sites generally show a linear function of Δ*G*_H_^ads^ apart from PPP dimer and fluorene. Δ*G*_4_ and Δ*G*_4_^a^ decrease with increasing Δ*G*_H_^ads^. An accurate BEP relation can also be derived for this step, based on the structural similarity between the initial, transition, and final states. The activation energy barrier is determined only by the formation of the H–H bond and the breaking of the C–H bond during H_2_ formation. The outliers are attributed to the structural differences between the initial and transition states (see Figures S7 and S8).

We next calculated the activity map, often known as the volcano plot^71^ of descriptors vs TOF due to its shape, with respect to the reaction rate (TOF), as a function of Δ*G*_H_^ads^ and Δ*G*_3-ref_ (Figure 2c and Table S8). It can also be observed that the rate is affected by different paths in different regions. In the triangle region with Δ*G*_3-ref_ > −0.75 eV and Δ*G*_H_^ads^ < 1.5 eV, the activity volcano results show that the Tafel-2P path is the RDS, and the rate of generation of H_2_ is determined by both Δ*G*_H_^ads^ and Δ*G*_3-ref_. For the remaining part of the activity map, the H^+^ adsorption path is the RDS, and the rate is determined only by Δ*G*_3-ref_. This region corresponds to the highest reactivity with Δ*G*_3-ref_ < −1 eV, where the activation energy of the RDS is low, and the free reaction energy is negative. Under these conditions, it can be concluded that the H^+^ adsorption and Tafel-2P steps are both fast enough. If the rate of production of P^•–^ (*R*_P^•–^_) is much slower, the HER rate is determined mainly by the generation of P^•–^. It should be noted that P2, P8–23, and P8–92 were near the highest activity region under standard conditions. The performance of the dimer molecules is lower than that of their monomers in our calculation because the Δ*G*_3-ref_ of the dimers is less negative and forms more PH^•^ than monomers.

The possible role of the small quantities of cocatalysts present in the sample is worth discussing. A residual concentration of Pd is often present in the system and is derived from the synthesis of conjugated polymers via Suzuki–Miyaura coupling.^29^ This is difficult to eliminate, and a significant effect of residual Pd-contaminated polymer on the HER activity was reported,^27−29^ indicating that residual metals may act as unintended active sites. However, the reactivity of some polymer materials showed no change after platinum deposition.^6^ Notably, advances in metal-free catalysts, including molecule^72−76^ and polymer^77−79^ catalysts, demonstrate that organic catalysts alone can effectively catalyze the HER. Therefore, it is important to elucidate the mechanisms of photocatalysis driven by organic components.

### 3.3 Screening Descriptors: Electronic Properties of the Selected Best-Known Polymer with High Activity

**Figure 3:**
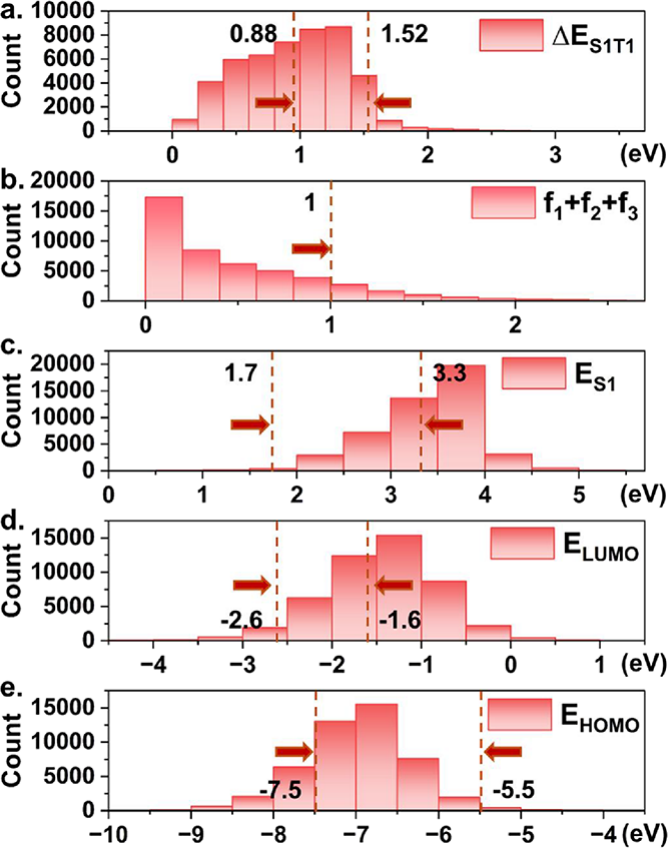
Histograms for the distribution of properties in the data set: (a) energy gap between S_1_ and T_1_ Δ*E*_S_1_T_1__, (b) sum of oscillator strengths of S_1_, S_2_, and S_3_, (c) optical gap *E*_S_1__, (d) LUMO energy *E*_LUMO_, and (e) HOMO energy *E*_HOMO_. Narrows show the range of values for the best-known catalysts.

We then identified potential catalysts with properties similar to those of the high-performance catalysts. This method has been successfully used in efficient and accurate high-throughput screening for “electronic copies” of the best organic solar cells and inverted singlet–triplet molecules that share the same known relevant characteristics.^47,80,81^ The properties of 8 polymers with best-performing HER, including P10, P38, P7, P8–92, PFODTBT, PCPDTBSO, P62, and P64, are listed as screening windows for identifying new catalysts, as shown in Table 2. As the initial step of the reaction, organic photocatalysts should have a solid ability to absorb visible light. This ability requires the minimum energy of the optical gap (*E*(S_1_)) to be greater than the reaction free energy of 1.23 eV^82−84^ to activate the reaction. At the same time, the maximum energy of the optical gap should be less than 3 eV^82−84^ to absorb a broad range of visible light values, but at least greater than 1.8 eV, considering the relaxation rate of the photocatalyst. The large optical gap ensures that the internal conversion (IC) rate is suppressed to a range of 10^7^ to 10^12^ s^–1^,^85^ allowing the electron transfer rate of the photocatalyst to exceed the IC rate. The data within this range of the optical gap constitute around 22% of our data (see Figure 3).

**Table 2:** Best-Known HER Polymers^a^^b^

	*E*_HOMO_ (eV)	*E*_LUMO_ (eV)	*E*_S_1__ (eV)	*f*_max_	*E*_T1_ (eV)	Δ*E*_S_1_T_1__ (eV)	SA	rate (μmol g^–1^ h^–1^)
P10	–7.17	–2.31	2.81	2.03	1.85	1.07	TEA	3260^b^
P38	–6.61	–1.90	2.73	3.4	1.84	0.95	TEA	5226^c^
P7	–6.92	–2.08	2.79	2.78	1.87	1.01	TEA	3680^d^
P8–92	–6.65	–1.86	2.81	3.68	1.9	0.98	TEA	9828^e^
PFODTBT	–5.83	–2.40	1.66	0.79	0.14	1.52	ascorbic acid	50 000^f^
PCPDTBSO	–6.04	–2.16	2.29	4.49	1.41	0.88	TEA	24 600^g^
P62	–6.82	–2.01	2.77	3.2	1.9	0.93	TEA	5202.6^e^
P64	–6.89	–2.08	2.76	3.2	1.88	0.94	TEA	6038.5^e^

a Structure optimizations were performed at the BLYP35/3-21g* level, and property calculations were performed at the M06-2X/3-21g* level. *E*_S_1__ and *E*_T_1__ were calibrated between calculations and experiment values using the equations: *E*_S_1__ (experiment) = 0.6562 × *E*_S_1__ (calculation) + 0.6866 and *E*_T_1__ (experiment) = 0.6916 × *E*_T_1__ (calculation) + 0.3463 with RMSE = 0.2460 eV and *R*^2^ = 0.91 for a set of 106 molecules.^48,86,87^

b Data from ref (88).

c Data from ref (31).

d Data from ref (89).

e Data from ref (6).

f Data from ref (32).

g Data from ref (90).

The oscillator strength values for the first three excited states, orbital energy of HOMO (*E*_HOMO_), orbital energy of LUMO (*E*_LUMO_), and the energy gap between S_1_ and T_1_ (Δ*E*_S_1_T_1__) are other screen descriptors for high light adsorption reactions, achieving slight energy loss and efficient energy conversion during light absorption.^80,91,92^ We further calculated the corresponding oscillator strengths for the S_1_, S_2_, and S_3_ states, relating to the absorption probability, for the selected known polymer photocatalysts. The maximum value of the oscillator strength is between 0.79 and 4.49 (see Table 2), accounting for around 17.5% of our data (see Figure 3). The Δ*E*_S_1_T_1__ of the selected photocatalysts is between 0.88 and 1.52 eV, representing 52.8% of our data (see Figure 3). *E*_HOMO_ and *E*_LUMO_ are, respectively, between −7.17 to −5.83 eV and −2.4 to −1.86 eV.

### 3.4 Screening Results

We utilized 8 descriptors to identify novel potential photocatalysts within the 48 182 molecules extracted from the CSD. The 8 descriptors selected were aligned with each step of the mechanism. The oscillator strengths (*f*) of S_1_, S_2,_ and S_3_ are related to the absorption probability of each state. The energy gap between S_1_ and S_0_ (*E*(S_1_)) was chosen to evaluate the (i) visible light absorption range, (ii) internal conversion rate, and (iii) activation capacity for the HER. The HOMO energy (*E*_HOMO_) and LUMO energy (*E*_LUMO_) are related to the thermodynamic favorability of electron/hole transfer between the catalysts and reactants. The electron transfer energy between the SA and polymer (Δ*G*_2_) is used to assess the electron separation ability. The energy difference between S_1_ and T_1_ (Δ*E*_S_1_T_1__) is used to assess the energy loss and efficient energy conversion during light absorption.^80,91,92^ Finally, we selected the free energy of H^+^ adsorption (Δ*G*_3-ref_) and the adsorption energy of the H atom (Δ*G*_H_^ads^) to identify catalysts with excellent performance in secondary reactions.

In addition to *f*, *E*(S_1_), *E*_HOMO_, *E*_LUMO_, and Δ*E*_S_1_T_1__, we also calculated electron transfer energy Δ*G*_2_ between TEA and the photocatalyst, the free energy of H^+^ adsorption energy (Δ*G*_3-ref_), and the adsorption energy of the H atom (Δ*G*_H_^ads^) at the B3LYP/6-31G(d,p) calculation level. The reference values for these properties are obtained for 8 known catalysts^93,94^ (see Table 2), which exhibited exceptional performance. Table 2 shows that the value ranges of these properties are *f*_max_ > 0.79, −7.17 eV < *E*_HOMO_ < −5.83 eV, −2.39 eV < *E*_LUMO_ < −1.87 eV, and 0.88 eV < *E*_S_1_T_1__ < 1.52 eV. To prevent missing optimal candidates, we expanded the range of searching conditions with respect to the known catalysts to 0.8 eV < *E*_S_1_T_1__ < 1.6 eV, −7.5 eV < *E*_HOMO_ < −5.5 eV, −2.6 eV < *E*_LUMO_ < −1.6 eV. The lowest excited state is imposed to be in the visible range, 1.7 eV < *E*_S_1__ < 3.3 eV, to match the maximum solar irradiation. The additional condition *f*_S1_ + *f*_S2_ + *f*_S3_ > 1 retains materials with the strongest light absorption in any of the 3 excited states considered in the original data set. Another descriptor is that the reaction energy of the electron transfer step should be thermodynamically favorable (Δ*G*_2_ < 0 eV). It should be noted that the key idea for selecting screening criteria is that they can be obtained from experimental best-in-class materials. The descriptors’ boundaries are not immutable but rather evolve with the change in such exemplary materials and can change as well as the descriptors that are chosen. One can use the information provided in SI to select materials using different boundaries

**Figure 4:**
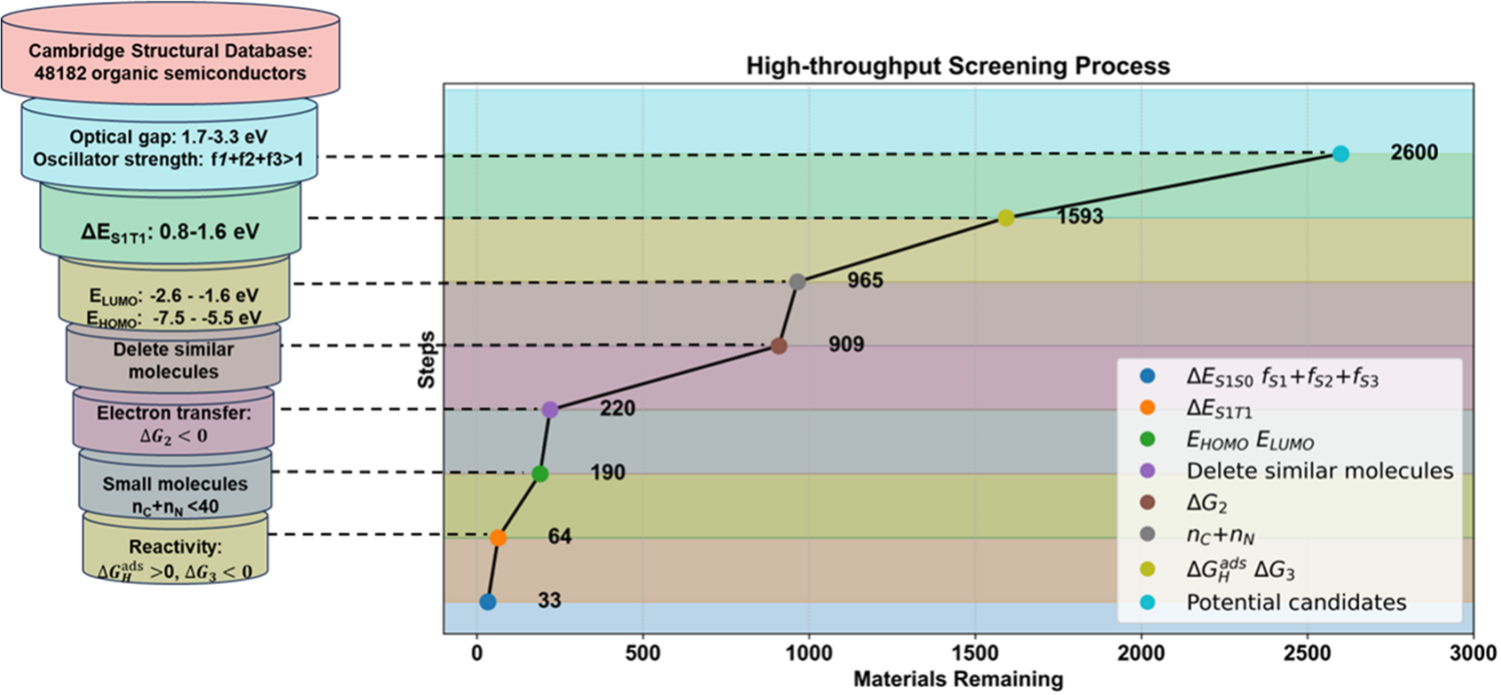
Steps for the screening process.

The detailed steps and results for the high-throughput screening can be summarized as follows (shown graphically in Figure 4):

1. The screening process efficiently narrowed the selection to 2600 molecules with promising light absorption ability based on their optical gap between 1.7 and 3.3 eV and oscillator strength *f*_S1_ + *f*_S2_ + *f*_S3_ > 1, with calculations exceeding the free energy of water splitting of 1.23 eV and being smaller than about 3 eV to adsorb solar radiation.^95^
2. After applying the condition of moderate *E*_S_1_T_1__ between 0.8 and 1.6 eV, which suppresses triplet recombination and acquires a small exciton dissociation driving force,^91^ 1593 molecules remained. This condition has been proven important for high-efficiency organic photovoltaics.^91^
3. 965 molecules remained under the conditions of −7.5 < *E*_HOMO_ < −5.5 and −2.6 < *E*_LUMO_ < −1.6 eV.
4. After the manual removal of similar molecules, 909 molecules remained. In this step, molecules have the same structure but different names (ID) or only have changes in one or two functional groups, such as alkyl and silyl groups, which cannot significantly affect their electronic and protonation properties.
5. 220 molecules remained with Δ*G*_2_ < 0.
6. 190 small molecules were identified for which the total number of C and N atoms was below 40.
7. Under the conditions of Δ*G*_H_^ads^ > 0 and Δ*G*_3-ref_ < 0, only 64 potential candidates remained.

This approach enabled the identification of 64 potentially new photocatalysts that will be explored next.

It should be noted that the calculations for steps 1, 2, and 3 were performed in ref (47).

**Figure 5:**
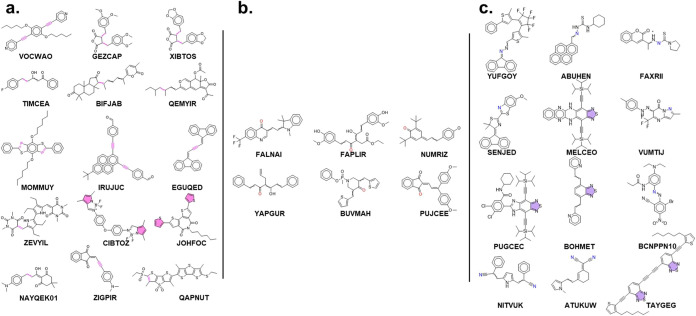
(a) Structures of the identified C-site photocatalysts with the top performance. (b) Structures of the identified N-site photocatalysts with the top performance. (c) Structures of the identified O-site photocatalysts with the top performance. The molecular labels used here are from the ID in CSD.

We further narrowed down the 64 candidates to 33 by considering the effects of the variety of active sites on the rate, including the C and N sites that were already used experimentally, as well as the novel site. We identified 46 C-site, 12 N-site, and 6 O-site molecules among the remaining 64 molecules. Our results demonstrated remarkable BEP linear relations (see Figure 3a) for C-type photocatalysts, leading to an effective way to estimate the activation energy. We further confirmed the computational accuracy based on the alignment trend between the experimental and calculated rates (see Figure S9). We thus can use the Δ*G*_3-ref_ and Δ*G*_H_^ads^ values to estimate their H_2_ production rates through an activity map. The results show that 28 molecules were predicted to have excellent secondary reaction performance at a TOF over 10^5^ s^–1^ (see Figure 3 and Table S8) on their active sites. Among these 28 potential molecules, we finally identified 15 carbon-type molecules with a TOF of about 10^7^ s^–1^, indicating a high expected performance comparable to that of known photocatalysts (see Figure 5a).

We discovered 4 N active site molecules with the same 2,1,3-benzothiadiazole group like PFODTBT (see Figure 5c). Even though we did not find a general trend for the N site because of the limited data size, we assumed that the molecules with the 2,1,3-benzothiadiazole group site could achieve high activity due to the experimentally verified high rates of PFODTBT and PCPDTBSO that rival the other best photocatalysts (see Table 2). It is reasonable to anticipate that candidates with this kind of active site may also exhibit notable performance in the HER, given their ability to adsorb H^+^ and experimental performance.^96^ In addition to traditional N- and C-site photocatalysts, we found two novel kinds of active site photocatalysts: (1) 8 novel N site-type molecules with a cyano group or imine group (see Figure 5c) and (2) 6 O-site molecules with a carbonyl group (see Figure 5b). These 14 novel-site kinds of photocatalysts will be reported in future studies.

We summarize the properties of 33 potential candidate molecules (in Tables 3 and S9). The *E*_S_1__ and *E*_T_1__ energies were calibrated to experimental data using established protocols^48,86,87^ with linear regression against the data for 106 molecules (fitting parameters in Table 3). The calibrated DFT/BLYP35/3-21G*and TD-DFT/M06-2X/3-21G* methods can reproduce the excitation energies with a root mean error of 0.246 eV (RMSE) and coefficient of determination *R*^2^ = 0.91. After calibration, the optical gap of selected molecules is narrowed to between 2.26 and 2.84 eV, satisfying the criteria of the primary reaction. The calibrated energy difference between S_1_ and T_1_ also fell within the search windows, ranging between 0.82 and 1.33 eV. We further utilized the energy gap law to estimate the internal conversion (IC) rates of the molecules, and most of their IC rates ranged from 10^8^ to 10^9^ s^–1^. Therefore, these molecules are expected to have a faster electron transfer rate than their IC rates. However, the FAXRII and BCNPPN10 molecules with the N site have the most considerable IC results at 10^10^ s^–1^. Considering the TOF results of the secondary reaction, it can concluded that C-site molecules are the most promising candidates. Molecules with N and O sites are assumed to react well in primary and thermodynamically favored secondary reactions, which still needs to be verified experimentally.

**Table 3:** Calibrated Descriptors for 33 Selected Potential Photocatalysts (Unit: Energy: eV, Rate: s^–1^)^a^

name	*E*_S_1__	*E*_T_1__	*E*_ST_	IC rate
VOCWAO	2.60	1.52	1.07	9.01
GEZCAP	2.64	1.50	1.13	8.87
XIBTOS	2.81	1.71	1.10	8.22
TIMCEA	2.71	1.90	0.82	8.57
BIFJAB	2.66	1.33	1.33	8.77
QEMYIR	2.75	1.53	1.22	8.44
MOMMUY	2.77	1.71	1.06	8.34
IRUJUC	2.84	1.81	1.03	8.09
EGUQED	2.72	1.63	1.09	8.54
ZEVYIL	2.45	1.47	0.99	9.56
CIBTOZ	2.81	1.70	1.10	8.22
JOHFOC	2.72	1.78	0.94	8.54
NAYQEK01	2.69	1.72	0.97	8.67
ZIGPIR	2.76	1.85	0.91	8.39
QAPNUT	2.65	1.76	0.89	8.82
FALNAI	2.56	1.45	1.12	9.14
FAPLIR	2.46	1.62	0.85	9.51
NUMRIZ	2.61	1.27	1.34	8.96
YAPGUR	2.47	1.65	0.82	9.49
BUVMAH	2.85	2.01	0.83	8.07
PUJCEE	2.80	1.73	1.07	8.24
YUFGOY	2.73	1.44	1.29	8.52
ABUHEN	2.72	1.46	1.26	8.54
FAXRII	2.26	1.18	1.09	10.29
SENJED	2.69	1.65	1.03	8.67
MELCEO	2.82	1.90	0.92	8.17
VUMTIJ	2.83	1.56	1.27	8.12
PUGCEC	2.71	1.58	1.14	8.57
BOHMET	2.72	1.66	1.06	8.54
BCNPPN10	2.29	1.36	0.93	10.16
NITVUK	2.75	1.67	1.09	8.42
ATUKUW	2.78	1.52	1.26	8.32
TAYGEG	2.54	1.63	0.92	9.21

a Structure optimizations were performed at the BLYP35/3-21g* level, and property calculations were performed at the M06-2X/3-21g* level. *E*_S_1__ and *E*_T_1__ were calibrated between calculations and experiment values using the equations *E*_S_1__ (experiment) = 0.6562 × *E*_S_1__ (calculation) + 0.6866 and *E*_T_1__ (experiment) = 0.6916 × *E*_T_1__ (calculation) + 0.3463 with RMSE = 0.2460 eV and *R*^2^ = 0.91 for a set of 106 molecules.^48,86,87^

## 4 Conclusions

We developed a fast HTVS method to screen the photocatalysts from 48 182 organic semiconductors using 8 descriptors. These descriptors include the primary reaction-related properties like the optical gap, oscillator strength, energy difference between S_1_ and T_1_, HOMO and LUMO energies, and the reaction energy of electron transfer, and secondary reaction-related properties like H^+^ adsorption and H–H coupling. We identified 33 organic molecules with distinct structures as potential photocatalysts, including 15 carbon-site photocatalysts, 4 2,1,3-benzothiadiazole nitrogen-site photocatalysts, 8 carbonyl nitrogen or imine nitrogen-site photocatalysts, and 6 carbonyl oxygen-site photocatalysts. After considering the calibrated properties, we further determined their potential performance. Even though carefully designed data-driven machine learning methods can incorporate physical descriptors, our approach provides a direct description through physically grounded microkinetics of the reaction and its similarity to existing compounds. The predictions were interpretable, controlled, and verified experimentally. Besides, the molecules identified in this study are, by construction, synthetically accessible and, therefore, suitable for experimental testing. Our research expands the possible range of organic photocatalysts, which is beneficial for developing more efficient polymer and molecular photocatalysts.

Although this work focuses on the reactivity of metal-free organic catalysts in the HER, the potential roles of trace Pd residues and other inorganic cocatalysts remain to be explored. Future mechanistic studies can be carried out to explore interfacial interactions through experiments and computational modeling.

## Data Availability

Coordinates of transition state structures (https://github.com/Lei2123/coordinate-files).

## Supporting Information Available

Detailed information on electron transfer energy, energetic pathways of the HER, structure information, and microkinetic method (PDF)
