# Supplementary material for: High-Throughput Screening of Molecule/Polymer Photocatalysts for the Hydrogen Evolution Reaction
Source: ACS Catal. 2025 Apr 10;15(9):6690–701. doi: 10.1021/acscatal.5c01785 (PMC12054366; doi:10.1021/acscatal.5c01785)
Supplement: Supplementary file 1 — cs5c01785_si_001.pdf [file cs5c01785_si_001.pdf]

Supporting information

High-throughput screening of molecules/polymer photocatalysts for Hydrogen evolution reaction

*Lei Shi, Alessandro Troisi**

A.Troisi@liverpool.ac.uk

Department of Chemistry, University of Liverpool, L69 7ZD, UK

SI. No	Contents	Page
**Table S1**	The molecule structures of the models	S5
**Table S2**	The electron transfer free energy ΔG_2 (eV) for trimers and monomers	S7
**Table S3**	The energy and free energy of the active site for the H^+ adsorption step	S8
**Figure S1.**	The structure for initial and transition state in the H^+ adsorption step.	S10
**Table S4**	The reaction-free energy and activation energy barrier for the H^+ adsorption step	S11
**Figure S2.**	The distance of the C-C bond and the distance between the best-fit planes of the protonated phenyl rings for initial states with the triplet state and transition states with the singlet state in the Tafel 2P path.	S12
**Figure S3.**	The free energy difference between the singlet states and triplet states for different molecules.	S14
**Figure S4.**	Schematic illustration of activation energy in the energy	S15

	diagram.	
**Figure S5**	The distance of the C-C bond and the distance between the best-fit planes of the protonated phenyl rings for initial states with singlet state in the Tafel 2P path	S16
**Table S5**	The energy and free energy for initial and transition states in the Heyrovsky path.	S17
**Table S6**	The structures for Initial states and transition states in the Tafel 1P path	S19
**Microkinetic model details**	**Microkinetic model details**	S21
**Table S7**	The free reaction energy, activation energy barrier (eV) and estimated rate TOF by BEP relation (TOF_{BEP} s^{-1}) and by direct calculation (TOF s^{-1}).	S24
**Figure S6**	**a.** Scaling relations of the descriptor of reaction 3 ($\Delta G_{3-\mathrm{ref}}$) vs reaction free energy (ΔG_3). **b.** Scaling relations of the descriptor of reaction 4 ($\Delta G_{\mathrm{H}}^{\mathrm{ads}}$) vs reaction free energy (ΔG_4).	S27

**Figure S7.**	Top and side view of the initial state (IS) and transition state (TS) structure for PHD.	S28
**Figure S8.**	Top view of the initial state (IS) and transition state (TS) structure for PPP-dimer, fluorene and PPP-4.	S29
**Table S8**	Calculated descriptors for 54 selected potential photocatalysts (unit: energy: eV, TOF: s^{-1})	S30
**Table S9**	Calculated descriptors for 33 selected potential photocatalysts (unit: energy: eV, TOF: s^{-1})	S33
**Figure S9**	**Figure S9**. The comparison between **a**. The experimental rate trend and **b**. The calculated rate trend for P10, P38, P7, P8-92 and PFODTBT. The calculated rates were obtained from calculated monomers. The discrepancies could be attributed to the residue Pd (0.1% Pd (w/w)) for PFODTBT	S35

Table S1 The molecule structures of the models

Name	Structures	Ref
poly(benzothiadiazole) (B-BT-1,4-E)	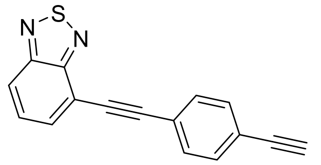	*ACS Energy Lett.* 2018, 3, 10, 2544-2549
PFODTBT	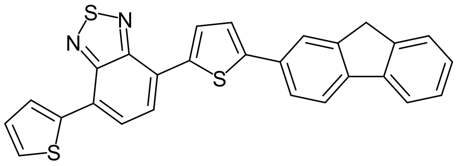	*Energy Environ. Sci.* 2017, 10, 1372-1376
poly(p-phenylene) (PPP-dimer)	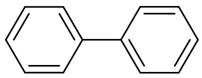	*J. Phy. Chem. 1990*, 94, 2068-2076
poly(p-phenylene) (PPP-mono)	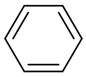	*J. Phy. Chem. 1990*, 94, 2068-2076
poly(p-phenylene) (PPP-4)	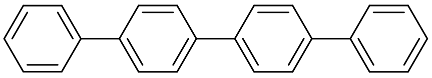	*J. Phy. Chem. 1990*, 94, 2068-2076
P2	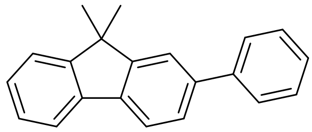	*J. Mater. Chem. A* 2021, 9, 19958-19964
P7	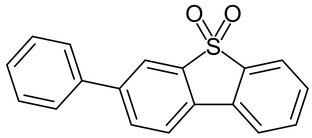	*J. Mater. Chem. A* 2021, 9, 19958-19964
P8-23	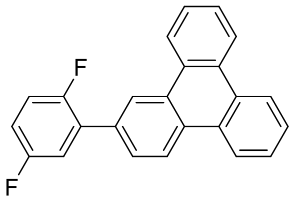	*J. Am. Chem. Soc.* 2019, 141, 22, 9063-9071
P8-92	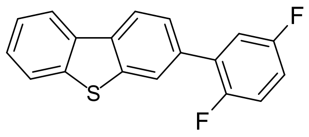	*J. Am. Chem. Soc.* 2019, 141, 22, 9063-9071
P10	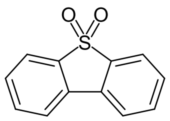	*J. Mater. Chem. A* 2021, 9, 19958-19964

P10-dimer	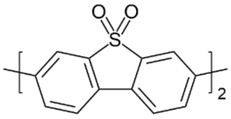	*J. Mater. Chem. A* 2021, 9, 19958-19964
P35	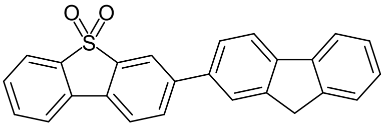	*J. Mater. Chem. A* 2021, 9, 19958-19964
P38	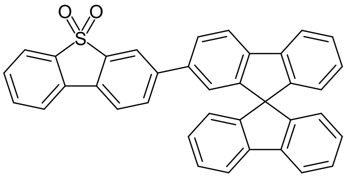	*J. Mater. Chem. A* 2021, 9, 19958-19964
P38-dimer	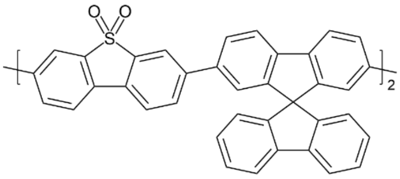	*J. Mater. Chem. A* 2021, 9, 19958-19964
Fluorene	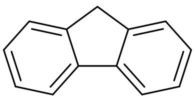	*J. Mater. Chem. A* 2019, 7, 22924.
PHD	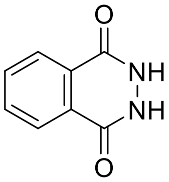	*Nano Res*. 2022, 15, 3835-3858.

Table S2 The electron transfer free energy ΔG_2 (eV) for trimers and monomers

	ΔG_2 (trimer)	ΔG_2 (monomer)
BBT-1,4-E	-0.9	-0.33
PFODTBT	-1.55	0.3
PPP	-0.64	-0.67
P2	-1.77	0.01
P7	-1.62	-0.51
P8-23	-1.7	-0.25
P8-92	-1.23	-0.33
P10	-1.39	-0.79
P35	-1.91	-0.2
P38	-2.64	-0.21
Fluorene	-0.79	-0.25
PHD	-0.80	-0.57

Table S3 The energy and free energy of the active site for the H^+ adsorption step

	Adsorption sites	$\Delta G_{\mathrm{H}}^{\mathrm{ads}}$ (eV)
BBT-1,4-E	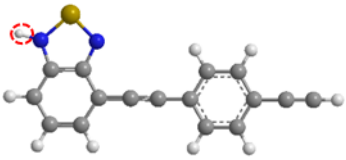	0.56
PFODTBT	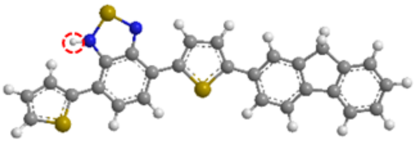	0.64
PPP-dimer	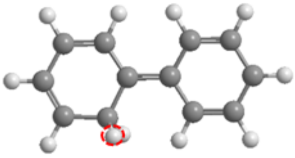	1.28
PPP-4	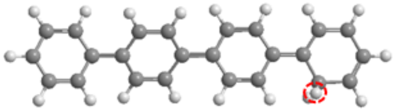	1.42
PPP-mono	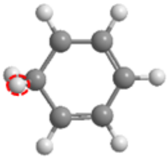	1.26
P2	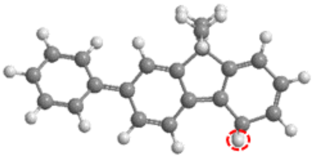	1.23
P7	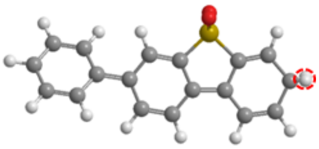	1.16
P8-23	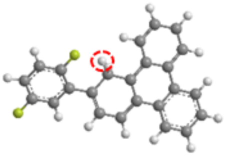	1.06
P8-92	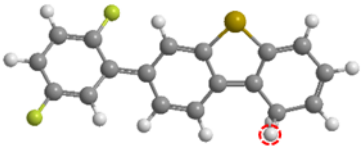	1.18

P10-dimer	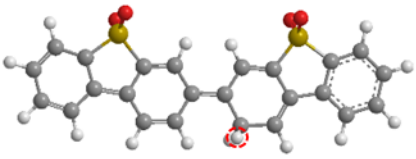	1.17
P10	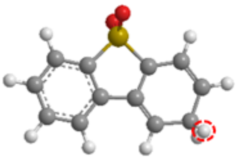	1.15
P35	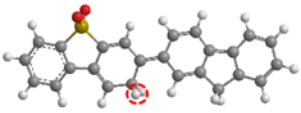	1.12
P38-dimer	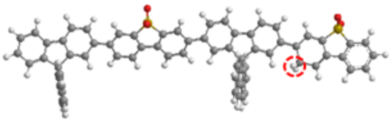	1.12
P38	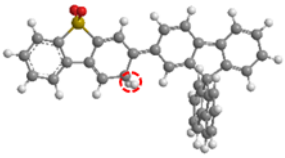	1.15
Fluorene	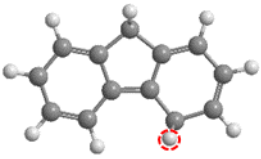	1.25
PHD	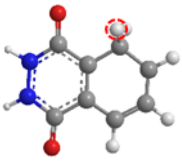	1.25
$H_9O_4^+$	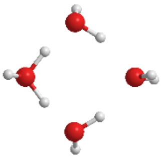	-
H_8O_4	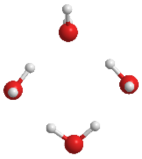	-
H_2		-

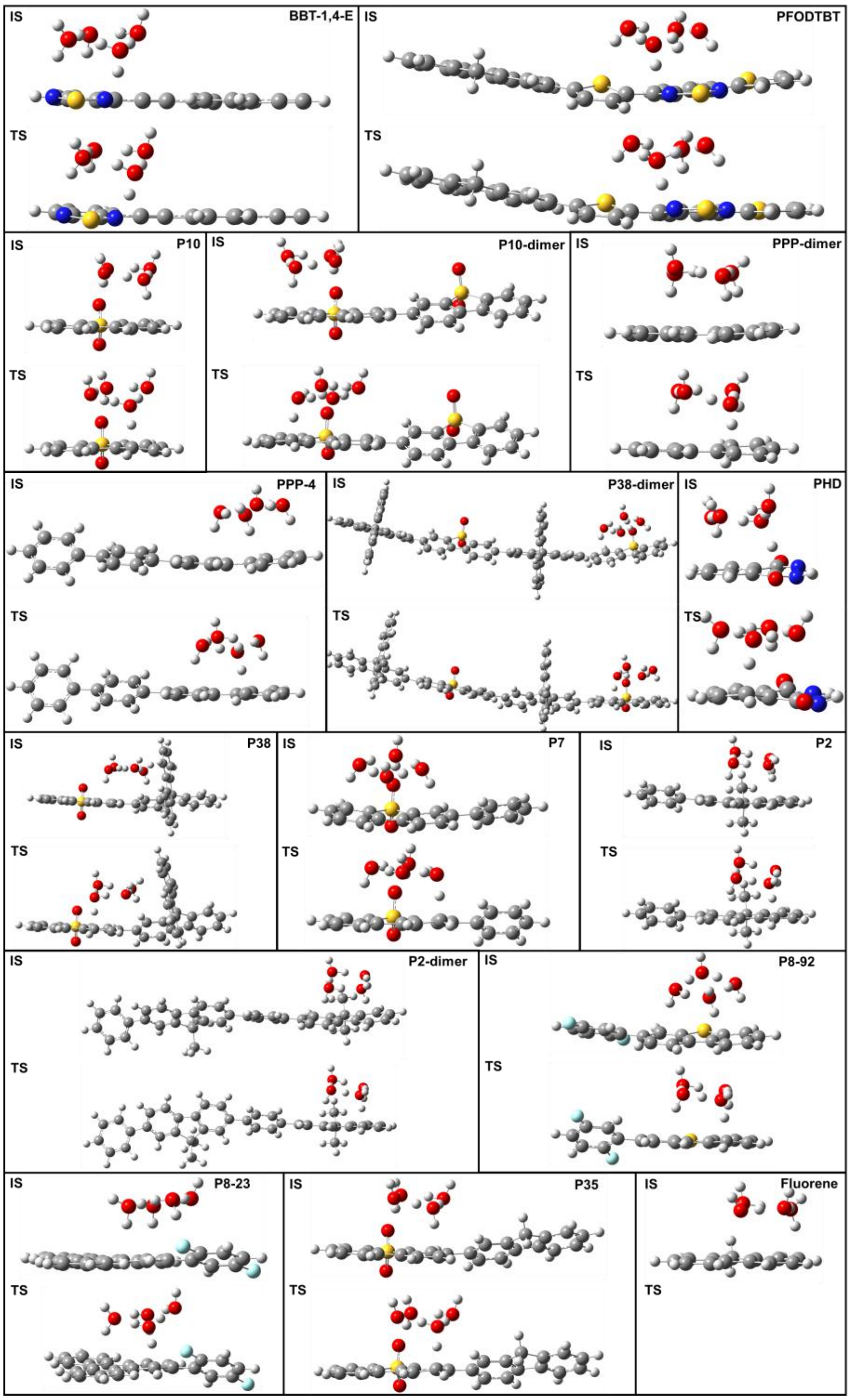

Figure S1. The structure for initial and transition states in the H^+ adsorption step, excluding fluorene and PPP-mono, whose transition state or initial state has not been located yet.

Table S4 The reaction-free energy and activation energy barrier for the H^+ adsorption step

Polymers	$\Delta G_{3-\mathrm{ref}}$(eV)	ΔG_3(eV)	ΔG_3^{a}(eV)
BBT-1,4-E	-0.6	-0.28	-0.05
PFODTBT	-0.47	-0.23	-0.08
PPP-mono	-	-	-
PPP-dimer	-1.44	-0.95	0.04
PPP-4	-0.99	-0.72	0.22
P2	-1.23	-1.09	0.07
P7	-0.57	-0.19	0.34
P8-23	-1.15	-0.85	0.03
P8-92	-1.03	-0.94	0.16
P10	-0.65	-0.17	0.41
P10-dimer	-0.28	-0.14	0.48
P35	-0.59	-0.24	0.24
P38	-0.58	-0.33	0.33
P38-dimer	-0.41	-0.08	0.49
PHD	-0.74	0.1	0.79
Fluorene	-1.59	-1.39	-

Note: -: the transition state has not been found for these species. The activation energy is obtained by $\Delta G_3^{\mathrm{a}} = G_{\mathrm{P^-\cdots H^+\cdots H_8O_4}} - G_{\mathrm{P^-+H_9O_4^+}}$. The activation energy barrier of B-BT-1,4-E (free energy correction: -0.06 eV) and PFODTBT (free energy correction: -0.11 eV) were confirmed by IRC.

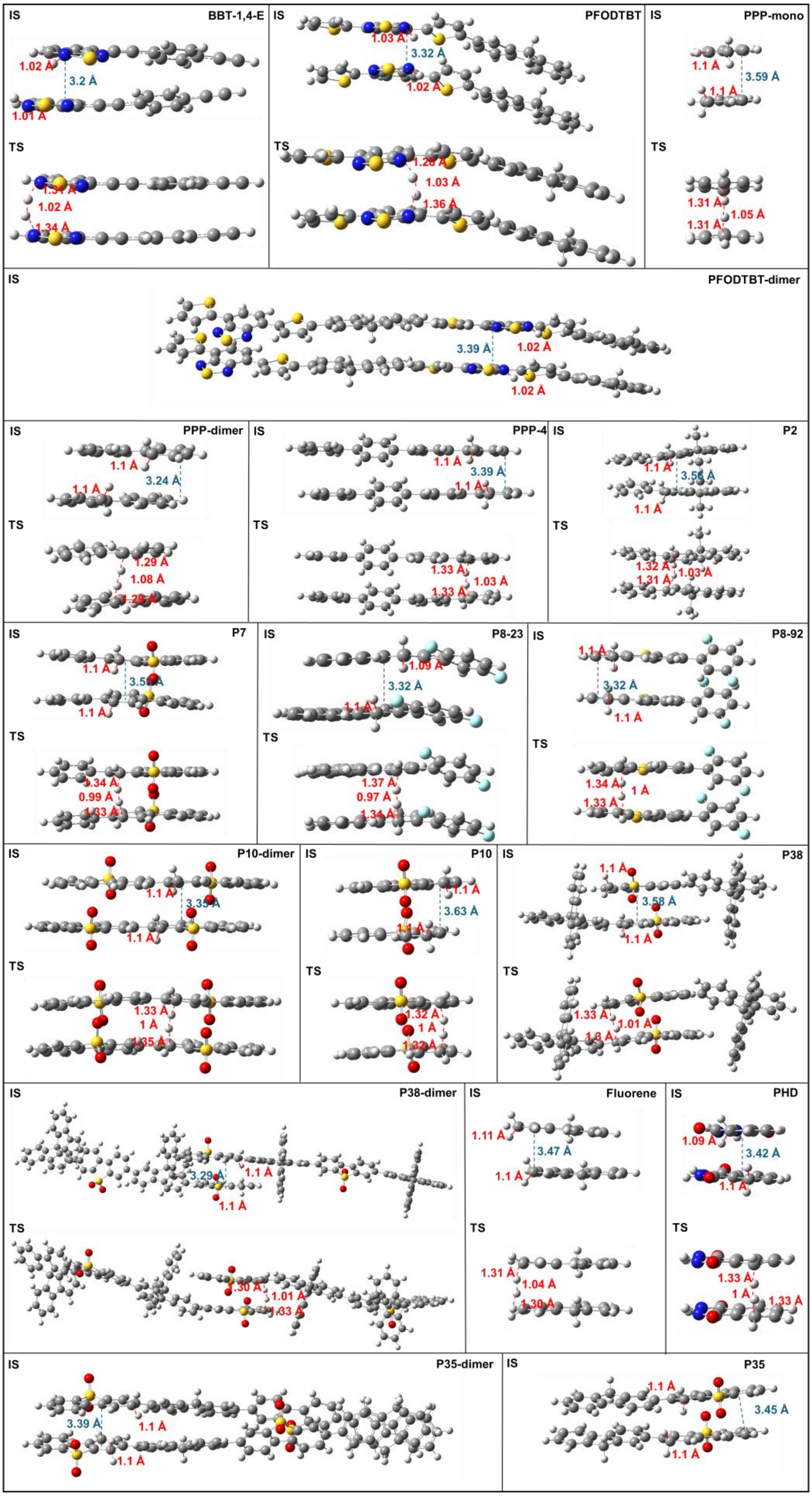

IS
BBT-1,4-E
1.02 Å
3.2 Å
1.01 Å
TS
1.02 Å
1.34 Å
IS
PFODTBT
1.03 Å
3.32 Å
1.02 Å
TS
1.03 Å
1.36 Å
IS
PPP-mono
1.1 Å
3.59 Å
1.1 Å
TS
1.31 Å
1.05 Å
1.31 Å
IS
PFODTBT-dimer
3.39 Å
1.02 Å
1.02 Å
IS
PPP-dimer
1.1 Å
3.24 Å
1.1 Å
TS
1.29 Å
1.08 Å
IS
PPP-4
1.1 Å
3.39 Å
1.1 Å
TS
1.33 Å
1.03 Å
1.33 Å
IS
P2
1.1 Å
1.1 Å
TS
1.32 Å
1.03 Å
1.31 Å
IS
P7
1.1 Å
1.1 Å
TS
0.99 Å
1.33 Å
IS
P8-23
1.09 Å
3.32 Å
1.1 Å
TS
1.37 Å
0.97 Å
1.34 Å
IS
P8-92
1.1 Å
3.32 Å
1.1 Å
TS
1.34 Å
1 Å
1.33 Å
IS
P10-dimer
1.1 Å
1.1 Å
TS
1.33 Å
1 Å
1.35 Å
IS
P10
1.1 Å
3.63 Å
1.1 Å
TS
1.32 Å
1 Å
1.32 Å
IS
P38
1.1 Å
3.58 Å
1.1 Å
TS
1.33 Å
1.01 Å
1.3 Å
IS
P38-dimer
3.29 Å
1.1 Å
1.1 Å
TS
1.30 Å
1.01 Å
1.33 Å
IS
Fluorene
1.11 Å
3.47 Å
1.1 Å
TS
1.31 Å
1.04 Å
1.30 Å
IS
PHD
1.09 Å
3.42 Å
1.1 Å
TS
1.33 Å
1 Å
1.33 Å
IS
P35-dimer
3.39 Å
1.1 Å
1.1 Å
IS
P35
1.1 Å
3.45 Å
1.1 Å

Figure S2. The distance of the C-H bond and the distance between the best-fit planes of the protonated phenyl rings for initial states with the triplet state and transition states with the singlet state in the Tafel 2P path, excluding P35 and PFODTBT-dimer (transition state has not been located yet).

It is noted that the transition state is dependent on the molecules' structure. Planar molecules like BBT-1,4-E, PFODTBT, PPP, P8-23, P8-92, and PHD can be parallel, like 'mirror symmetry'. The two H^+ can thus adsorb on the same site on these molecules. For others, especially the molecules with sulfone units, they are parallel like 'inversion symmetry'. The H^+ thus needs to adsorb on the different sites of these molecules with a short length to form H_2, which means one H may not be on their most favoured sites. The difference of $\Delta G_{\mathrm{H}}^{\mathrm{ads}}$ on the same benzene of two H adsorption sites of P10, P2, P7, and P38 is found as small as 0, 0, 0.01 eV and 0.05 eV, respectively. Thus, one can roughly neglect the effect of different sites on H-H bond formation probability.

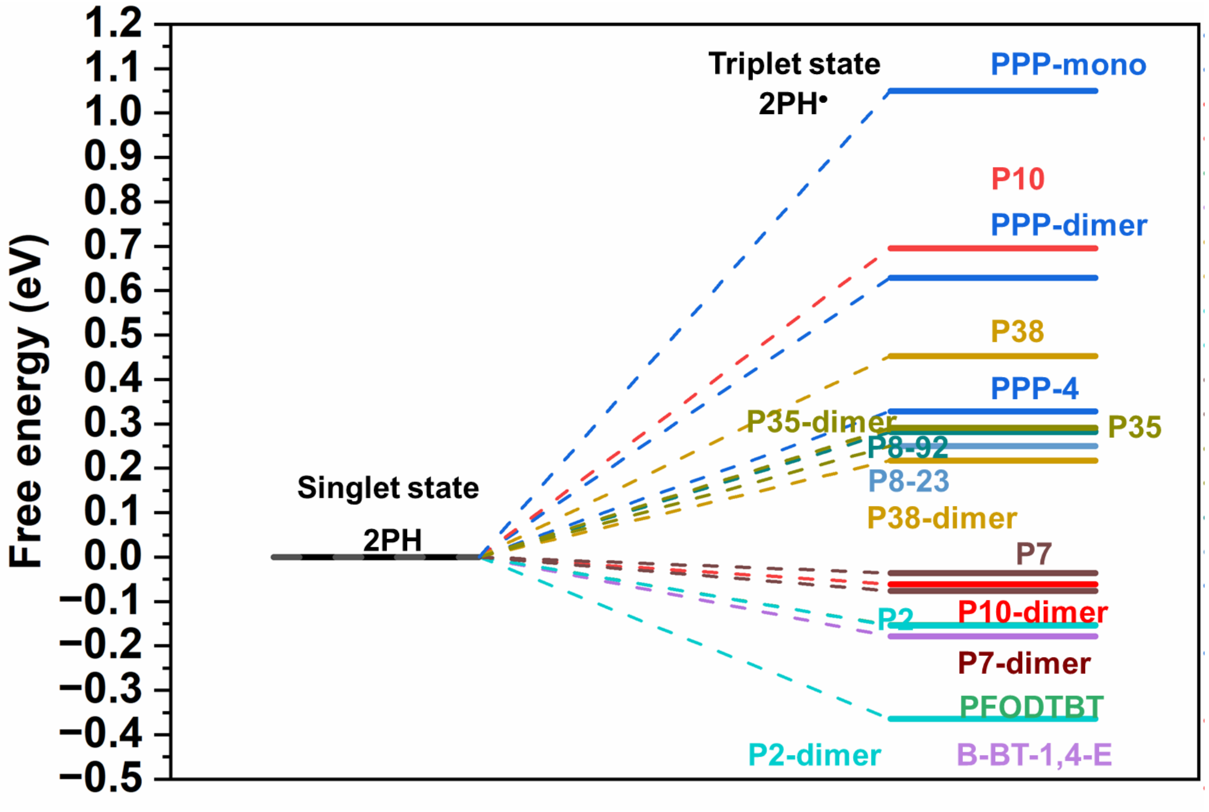

Figure S3. The free energy difference between the singlet states and triplet states for different molecules.

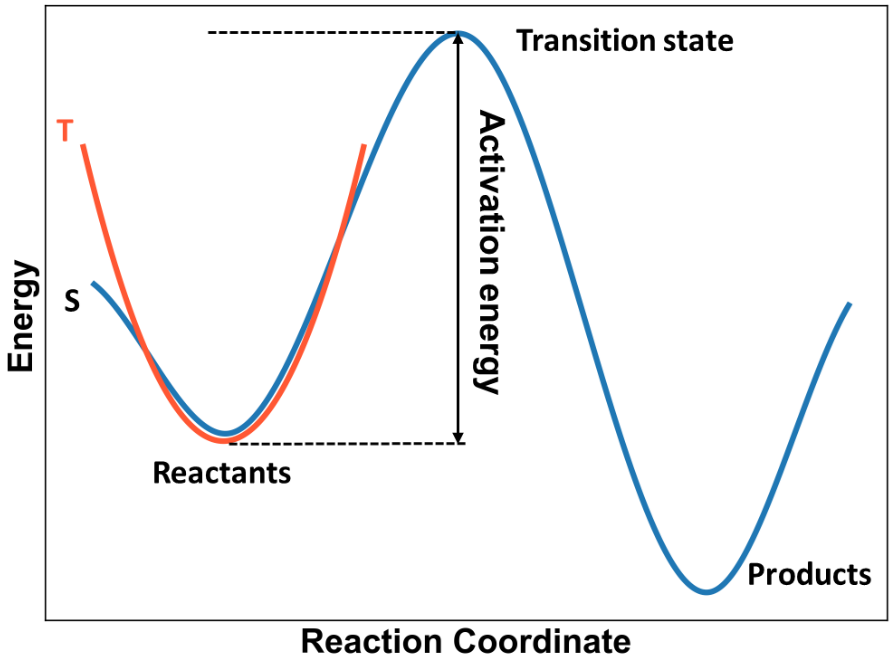

Figure S4. Schematic illustration of activation energy in the energy diagram.

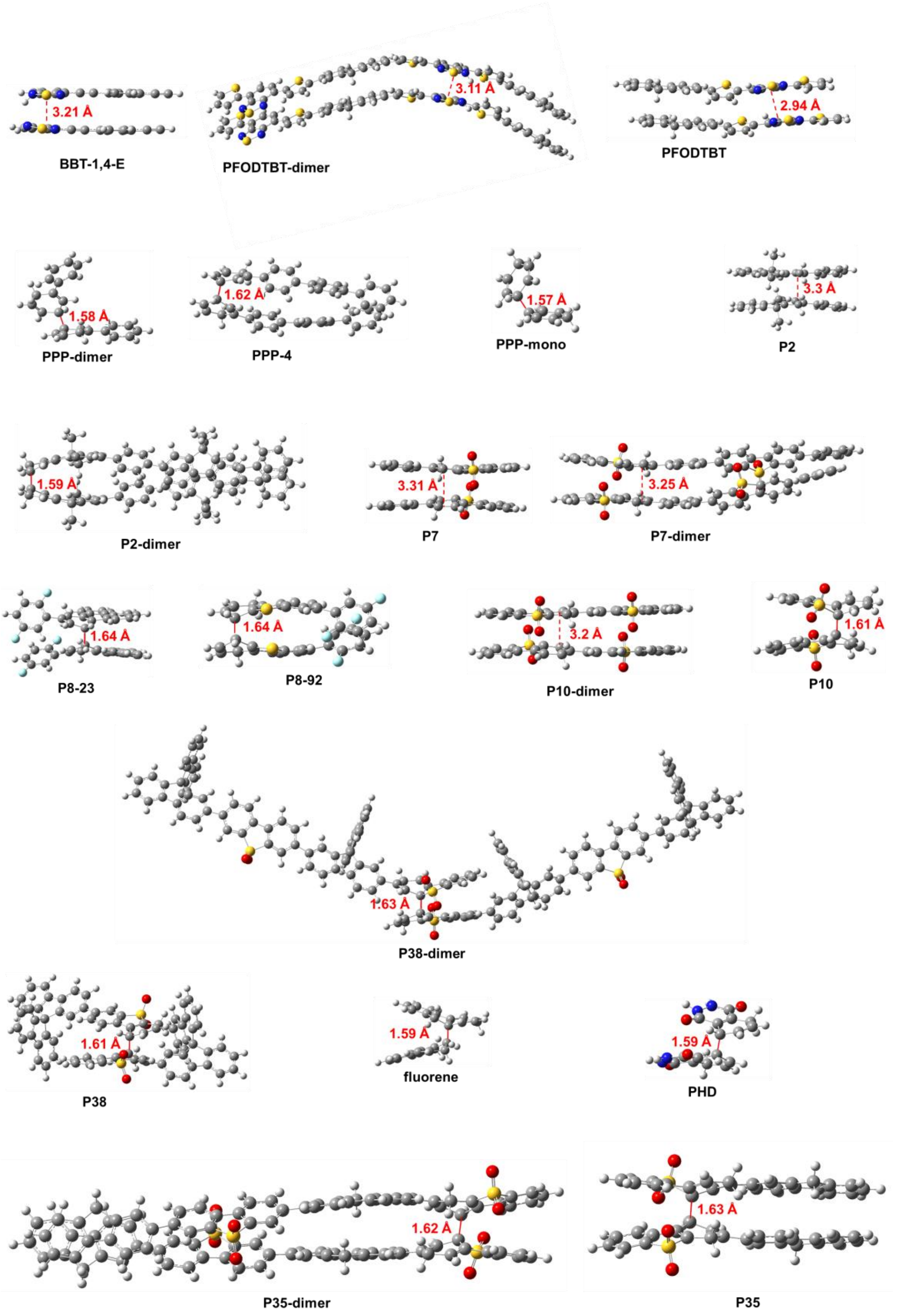

Figure S5 The distance of the C-C bond of the protonated phenyl rings for initial states with singlet state in the Tafel 2P path.

Table S5 The energy and free energy for initial and transition states in the Heyrovsky path.

	Initial state	Transition state
B-BT-1,4-E	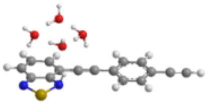	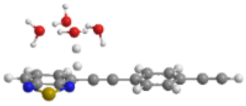
PFODTBT	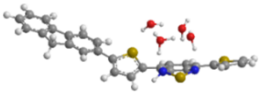	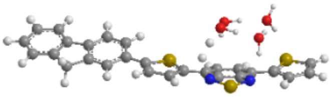
PPP-dimer	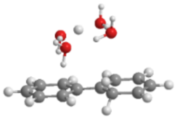	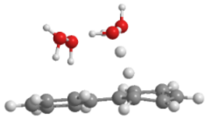
PPP-4	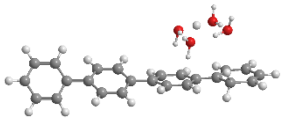	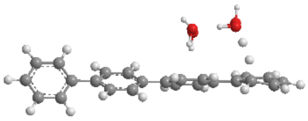
P2	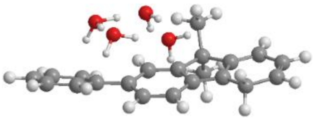	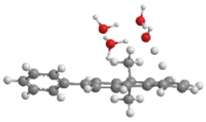
P7	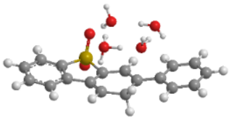	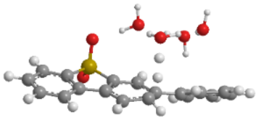
P8-23	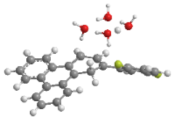	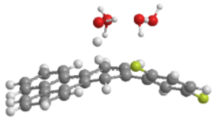
P8-92	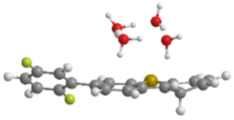	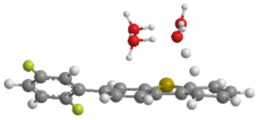

P10-dimer	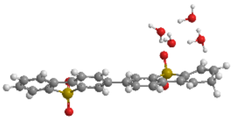	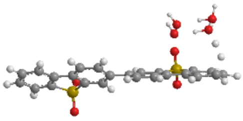
P10	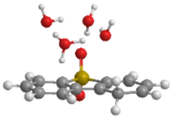	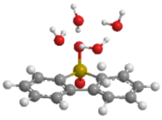
P38-dimer	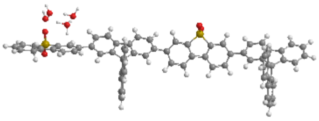	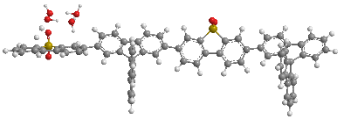
P38	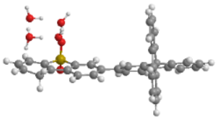	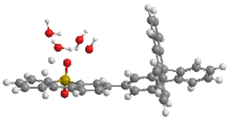
P35	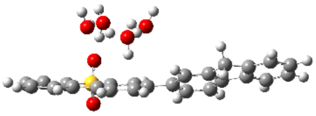	-
fluorene	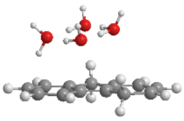	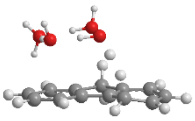
PHD	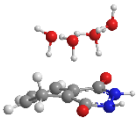	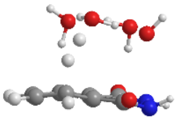

Note: transition state and initial state of PPP-mono and P35 have not located yet.

Table S6 The structures for Initial states and transition states in the Tafel 1P path.

	Initial state	Transition state
BBT-1,4-E	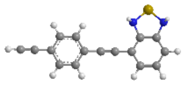	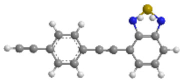
PFODTBT	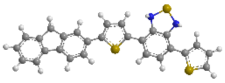	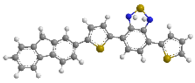
PPP-dimer	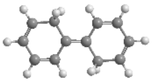	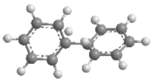
PPP-4	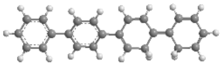	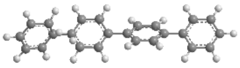
P2	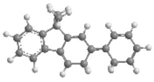	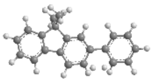
P7	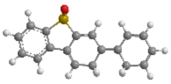	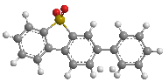
P8-23	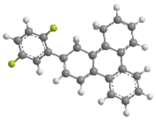	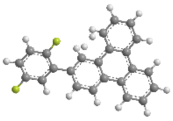
P8-92	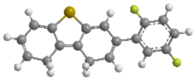	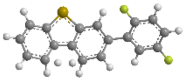
P10-dimer	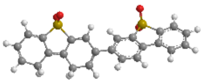	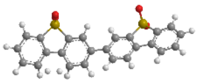
P10	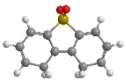	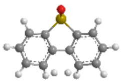
P38-dimer	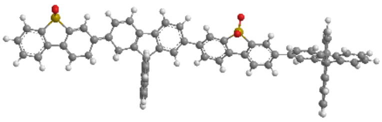	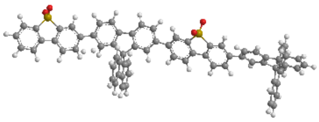
P35	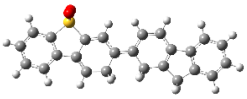	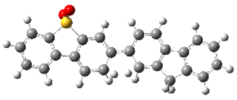

P38	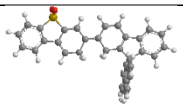	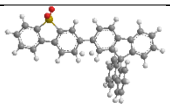
Fluorene	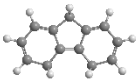	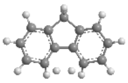

Note: The PPP-mono is excluded here as its transition state has not been located yet.

Microkinetic model details:

The concentration of each species (C_i) is assumed to be independent of time ($\frac{dC}{dt} = 0$) known as steady state assumption, and the rate-determining step (RDS) is assumed to be slow enough (R=R_{RDS}) so that other steps have already reached the equilibrium state (R_i=0). At the mean-field approximation[1], all adsorbed species are assumed to be distributed randomly with low interaction between reactants. The reaction rate of the elementary step at one active site of one compound is thus proportion to the product of the rate constant and probability of finding one reactant adjacent to another reactant, which is proportional to the concentration of each species.[2] The relation between forward and backward rate constant k^+ and k^-, and the equilibrium constant K of each step can be expressed as an Arrhenius expression:

$$k_n^+ = \frac{k_b T}{h} exp\left(\frac{-\Delta G_{\mathrm{n}}^{\mathrm{a}}}{k_b T}\right) \ (\mathrm{n} = 3{,}4) \tag{S1}$$

$$K_n = exp(\frac{-\Delta G_{\mathrm{n}}}{k_b T}) \ (n = 3{,}4) \tag{S2}$$

$$k_n^- = \frac{k_n^+}{K_n} \ (n = 3{,}4) \tag{S3}$$

The molecule catalysts in our model are assumed to be able to adsorb only one H^+ ion because the subsequent reaction is thermodynamically unfavourable because of the influence of the absorption of secondary photons. Besides, the strong adsorption energy of H^+ adsorbates makes it difficult for adsorbates to transfer from one active site to another. The rate of the elementary steps 3 and 4 is thus proportion to the concentration of each species, and the reaction rate equation of the elementary step (3) and step (4) is expressed as:

$$R_3 = k_3^+ C_{\mathrm{P}^{\bullet -}} * C_{\mathrm{H}^+} - k_3^- C_{\mathrm{PH}^{\bullet}} \tag{S4}$$

$$R_4 = k_4^+ C_{\mathrm{PH}^{\bullet}}^2 - k_4^- C_{\mathrm{P}}^2 P_{\mathrm{H}_2} \tag{S5}$$

If step 3 is the rate-determining step:

$$R_{RDS} = R_3 \tag{S6}$$

$$R_4 = 0$$

If step 4 is the rate-determining step:

$$R_{RDS} = R_4 \tag{S7}$$

$$R_3 = 0$$

The activation energy barrier and reaction energy can be further replaced by an equation obtained from the linear relation:

$$\Delta G_3^{\mathrm{a}} = 0.38 * \Delta G_{3-\mathrm{ref}} + 0.54 \tag{S8}$$

$$\Delta G_3 = 1.03 * \Delta G_{3-\mathrm{ref}} + 0.32 \tag{S9}$$

$$\Delta G_4^{\mathrm{a}} = -0.68 * \Delta G_{\mathrm{H}}^{\mathrm{ads}} + 1.21 \tag{S10}$$

$$\Delta G_4 = -1.69 * \Delta G_{\mathrm{H}}^{\mathrm{ads}} - 0.38 \tag{S11}$$

It is noted that the activation energy can be estimated to be a negative value by using the linear relation. If $\Delta G_3^a < 0$ and $\Delta G_4^a < 0$, the rate constant is determined by reaction energy.

If $\Delta G_3 > 0$, reaction energy is activated by ΔG_3, the R_3 can be evaluated by:

$$R_3 = \frac{k_b T}{h} exp\left(\frac{-\Delta G_3}{k_b T}\right) * \left(C_{\mathrm{P}^{\bullet-}} * C_{\mathrm{H}^+} - \frac{C_{\mathrm{PH}}}{K_3}\right) \tag{S12}$$

The concentration of PH$^\bullet$ can be solved by R_4=0:

$$C_{\mathrm{PH}^\bullet} = \sqrt{k_4^- C_{\mathrm{P}}^2 P_{\mathrm{H}_2} / k_4^+} \tag{S13}$$

If $\Delta G_3 < 0$, the rate constant is expected to be $k = \frac{k_b T}{h}$, the R_3 can be evaluated by:

$$R_3 = \frac{k_b T}{h} (C_{\mathrm{P}^{\bullet-}} * C_{\mathrm{H}^+} - C_{\mathrm{PH}}/K_3) \tag{S14}$$

If $\Delta G_4 > 0$, the R_4 can be evaluated by:

$$R_4 = \frac{k_b T}{h} exp\left(\frac{-\Delta G_4}{k_b T}\right) (C_{\mathrm{PH}^\bullet}^2 - C_{\mathrm{P}}^2 P_{\mathrm{H}_2}/K_4) \tag{S15}$$

The concentration of PH$^\bullet$ can be solved by R_3=0:

$$C_{PH^\bullet} = k_3^+ C_{P^{\bullet-}} * C_{H^+}/k_3^- \quad (S16)$$

If $\Delta G_4 < 0$, the rate constant is expected to be $k = \frac{k_b T}{h}$, the R_4 can be evaluated by:

$$R_4 = \frac{k_b T}{h}(C_{PH^\bullet}^2 - \frac{C_P^2 P_{H_2}}{K_4}) \quad (S17)$$

We then developed a microkinetic model of HER at conditions of standard condition[3] (C_{H^+} = 1 mol/L, C_{SA} = 1 mol/L, T= 298.15 K, P_{H_2} = 1 bar) at C active sites. The initial concentration of P can be assumed to be 1 mol/L. The concentration of catalysts accepting an electron from TEA in the excited state ($C_{P^{\bullet-}}$) should be lower than C_{SA}, and the overall efficiency of forming $P^{\bullet-}$ could be assumed at 0.1:

$$C_{P^{\bullet-}} \leq C_{SA} \quad (S18)$$

The possible concentration of $P^{\bullet-}$ is predicted to be 0.1 mol/l. We calculated the TOF (see **Table 7** TOF) of HER of monomers and some dimers for 11 polymers on C active sites. It can be seen that the TOF on the C site is higher than on the N site because the RDS are different. The value of the activation barrier of the H^+ adsorption step determines the reactivity on the C site (ΔG_3^a). The reactivity of the secondary reaction on P8-23, PPP-dimer, and P2 is predicted to be higher than those of other catalysts due to the lower activation energy barrier (ΔG_3^a). In contrast, the reactivity on N sites is predicted to be relatively low as the considerable activation energy of the RDS of the H-H coupling step (ΔG_4^a).

Next, we estimated TOF_{BEP} using BEP relation (see T**able S7**). An acceptable difference within around 2 orders of magnitude exists between the calculated TOF (using calculated activation energies by DFT) and estimated TOF_{BEP} (using the activation energies by BEP relation). One can conclude that the TOF of the secondary reaction on the C sites can be estimated by ΔG_H^{ads} and ΔG_{3-ref}. Besides, the monomers show higher activity than their dimers because of the lower activation energy of RDS.

Table S7 The free reaction energy, activation energy barrier (eV) and estimated rate TOF by BEP relation (TOF_{BEP} s^{-1}) and by direct calculation (TOF s^{-1}).

Polymers	$\Delta G_{3-\mathrm{ref}}$	$\Delta G_{\mathrm{H}}^{\mathrm{ads}}$	log (TOF_{BEP} /s)	log (TOF /s)
BBT-1,4-E	-0.6	0.56	-	-5.26
PFODTBT	-0.47	0.64	-	-2.39
PPP-dimer	-1.44	1.28	11.91	11.12
PPP-4	-0.99	1.42	9.02	8.07
P2	-1.23	1.23	10.57	10.61
P7	-0.57	1.16	6.33	6.05
P8-23	-1.15	1.06	10.05	11.29
P8-92	-1.03	1.18	9.28	9.09
P10	-0.65	1.15	6.84	4.86
P10-dimer	-0.28	1.17	2.72	3.68
P35	-0.59	1.12	-	6.46
P38	-0.58	1.15	6.46	6.72
P38-dimer	-0.41	1.12	6.39	3.51
PHD	-0.74	1.25	-	-1.56
Fluorene	-	1.42	-	-

-: not found for these species.

Computation of free energies

The Gibbs free energy of molar molecule to describe chemical processes at temperature T and pressure P can be obtained from electronic structure calculations under certain assumptions. This quantity is defined by:

$$G = E + PV - TS \tag{1.34}$$

where S and V are the entropy and volume. The internal energy E is obtained as follows:

$$E = E_{electron} + E_{ZPE} + E_{translation} + E_{rotation} + E_{vibration} \tag{1.35}$$

In equation (1.35), $E_{\text{translation}}$, E_{rotation}, and $E_{\text{vibration}}$ are the contributions of molar molecule translation, rotation and vibration to the internal energy. E_{electron} is the molar electronic energy, which refers to a local minimum energy of potential energy surface. The molar zero-point energy (E_{ZPE}) for molecules under the harmonic assumption is usually included in the calculation of E to estimate ground state energy:

$$E_{ZPE} = R\sum_{i=1}^{k}\frac{1}{2}hv_i \tag{1.36}$$

where k, v_i and R is vibrational modes, vibration frequencies and molar gas constant, 3N-6 is for nonlinear molecules, and 3N-5 is for linear molecules. The $E_{\text{translation}}$, E_{rotation}, and $E_{\text{vibration}}$ can be given as follows:

$$E_{translation} = \frac{3}{2}RT \tag{1.37}$$

$$E_{rotation}(linear) = RT \tag{1.38}$$

$$E_{rotation}(nonlinear) = \frac{3}{2}RT \tag{1.39}$$

$$E_{vibration} = RT\sum_{i=1}^{k}\frac{hv_i/k_B}{e^{hv_i/k_BT}-1} \tag{1.40}$$

The contribution of molar entropy includes molar electron (S_{electron}) transition ($S_{\text{translation}}$), rotation (S_{rotation}), and vibration ($S_{\text{vibration}}$) entropy:

$$S = S_{electron} + S_{translation} + S_{rotation} + S_{vibration} \tag{1.41}$$

The S_{electron} can be given as follows:

$$S_{electron} = R\ln(g_0) \tag{1.42}$$

where g_0 is the degeneracy of the ground state. The $S_{\text{translation}}$, S_{rotation}, and $S_{\text{vibration}}$ can be expressed as:

$$S_{translation} = R\left[\ln\left(\frac{(2\pi mk_BT)^{\frac{3}{2}}}{h^3}\frac{k_BT}{P}\right)+\frac{5}{2}\right] \tag{1.43}$$

$$S_{rotation}(linear) = R\left[\ln\left(\frac{8\pi^2 I(k_BT)}{\sigma h^2}\right) + 1\right] \quad (1.44)$$

where I is the moment of inertia and σ is the rotational symmetry number.

$$S_{rotation}(nonlinear) = R\left[\ln\left(\frac{8\pi^2\sqrt{8\pi^3 I_x I_y I_z}(k_BT)^{\frac{3}{2}}}{\sigma h^2}\right) + \frac{3}{2}\right] \quad (1.45)$$

where I_x, I_y, and I_z are moments of inertia on different axes.

$$S_{vibration} = R\sum_k [\frac{\frac{hv_i}{k_B}T}{e^{\frac{hv_i}{k_B}T} - 1} - \ln(1 - e^{\frac{hv_i}{k_B}T})] \quad (1.46)$$

The contribution of Gibbs free energy gas species such as H_2 can be described in a more accurate way using the empirical Shomate equation,[4] which can be expressed as:

$$G = E + \int_o^T C_P dT - TS \quad (1.47)$$

where the C_P is heat capacity. The C_P and S can be estimated using the Shomate equation. The Shomate equation was used to calculate the Gibbs free energy of H_2.

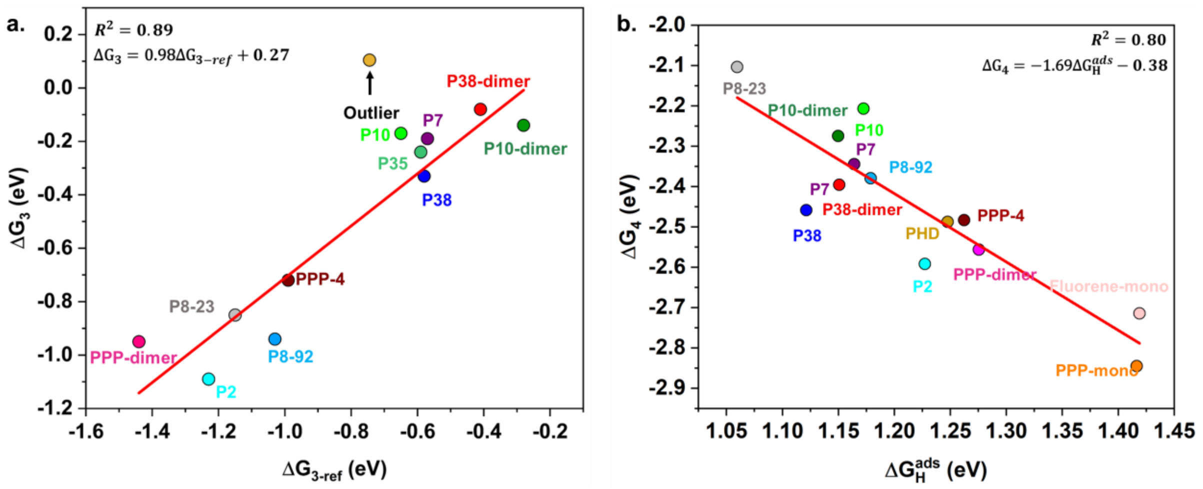

Figure S6 a. Scaling relations of the descriptor of reaction 3 ($\Delta G_{3-\mathrm{ref}}$) vs reaction free energy (ΔG_3). **b.** Scaling relations of the descriptor of reaction 4 ($\Delta G_{\mathrm{H}}^{\mathrm{ads}}$) vs reaction free energy (ΔG_4).

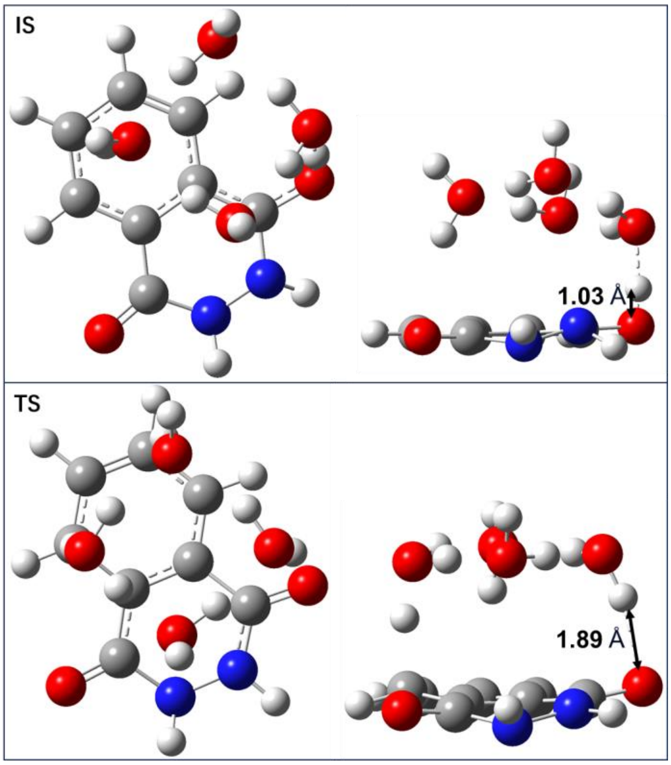

Figure S7. Top and side view of the initial state (IS) and transition state (TS) structure for PHD.

Figure S7 shows the initial structure and transition structure of the PHD. One can see the Hydrogen bond length difference between water clusters and the PHD (the side O atom). This distance increased from 1.03 Å to 1.89 Å during the transition state formation, indicating that this molecule's calculated activation energy is due to forming and breaking bonds between C-H-O and the hydrogen bond on the opposite side. Consequently, the PHD does not follow the BEP trend well and was excluded from **Figure 2 and S6**. In contrast, other molecules without additional bond formation follow the trend well (structure details see **Table 5**).

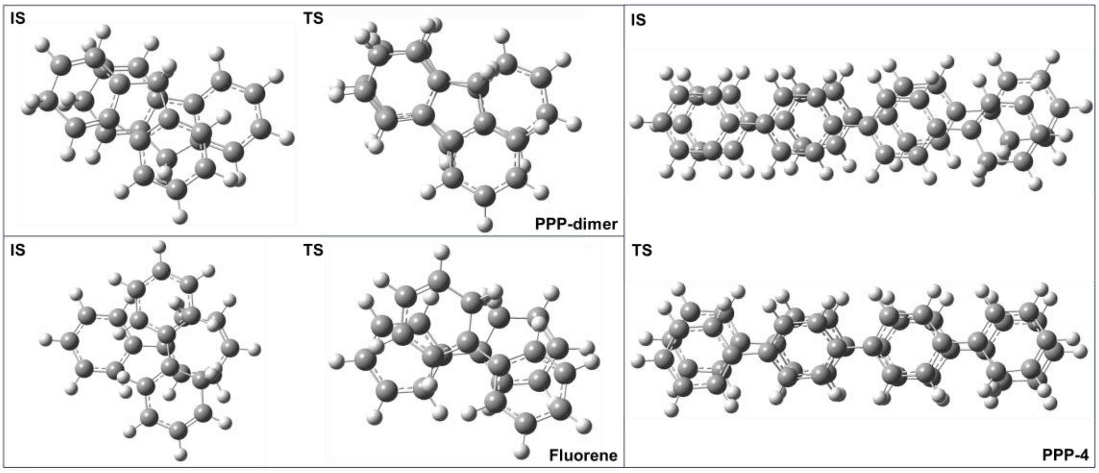

Figure S8. Top view of the initial state (IS) and transition state (TS) structure for PPP-dimer, fluorene and PPP-4.

The observed deviations of the activation energy barrier for PPP-dimer and fluorene can be attributed to the structure differences between their initial and the transition states, mainly due to the displacement of relative positions of molecules. **Figure S8** shows the plots' corresponding structures of outliers, including PPP-dimer and fluorene. One can see a distinct displacement between structures in these two molecules' initial and transition states for PPP-dimer and fluorene, resulting in a deviation from the BEP relation. In contrast, molecules like PPP-4 exhibit only slight displacement between their initial and transition states, thereby adhering more closely to the BEP trend. The additional structure information, including all molecules, can be found in **Figure S2**. The molecules with strong interaction energy (such as solid π stacking) between two molecules might follow the BEP trend because of the slight structure difference between the two states. It is noted that the N-site type molecules are excluded here due to the limited number of data sets available.

Table S8 Calculated descriptors for 54 selected potential photocatalysts (unit: energy: eV, TOF: s^{-1})

Name	log(TOF)	$\Delta G_{\mathrm{H}}^{\mathrm{ads}}$	$\Delta G_{3-\mathrm{ref}}$
VOCWAO	10.06	0.37	-1.16
GEZCAP	9.60	0.12	-1.10
XIBTOS	9.45	0.16	-1.08
TIMCEA	9.43	0.37	-1.07
BIFJAB	9.34	0.12	-1.06
QEMYIR	8.91	0.10	-1.00
MOMMUY	8.16	0.47	-0.89
IRUJUC	7.70	0.40	-0.83
EGUQED	7.53	0.27	-0.80
ZEVYIL	7.23	0.32	-0.76
CIBTOZ	7.21	0.76	-0.76
JOHFOC	7.19	0.42	-0.76
NAYQEK01	7.18	0.62	-0.75
ZIGPIR	7.15	0.35	-0.75
QAPNUT	6.99	0.32	-0.73
IXUTEB	6.85	0.67	-0.71
RAFHUD	6.66	0.56	-0.68
KARZIO	6.50	0.32	-0.66
FUQZEY	6.32	0.65	-0.63
HOSVET	5.87	0.34	-0.63
NEQNUU	5.20	0.44	-0.57
DUNVIR01	5.82	0.66	-0.56
CIVYAK	5.81	0.59	-0.56
ICOBOS	3.73	0.38	-0.55
GUZJAO	2.62	0.29	-0.54

GIHPOF	5.62	0.62	-0.54
CUSNEK	5.61	0.82	-0.53
NOZPOK	2.33	0.33	-0.52
MUBQOS	4.37	0.52	-0.52
WULVEG	5.41	0.65	-0.51
WIMHEH	5.38	0.67	-0.50
LEVGOK	1.76	0.39	-0.49
MUDSIQ	5.26	0.95	-0.48
WULVEG	5.41	0.65	-0.51
WIMHEH	5.38	0.67	-0.50
LEVGOK	1.76	0.39	-0.49
MUDSIQ	5.26	0.95	-0.48
UFUVEZ	5.06	0.95	-0.46
ZUCBUX	3.99	0.73	-0.43
KOVGAF	-2.14	0.20	-0.43
HARHIT	2.81	0.67	-0.42
ZEVYEH	-1.29	0.32	-0.42
SELRIM	0.37	0.48	-0.41
HOHYOV	4.39	0.84	-0.41
KOXFIP	3.08	0.81	-0.38
MUBCES	-0.87	0.49	-0.37
VIZLOF	1.76	0.73	-0.37
DAWJIX	2.38	0.79	-0.37
BACVUA	-4.63	0.21	-0.35
EPOHEY	0.00	0.70	-0.32
ETGLAU01	-4.41	0.40	-0.29
OBEQEU	-1.44	0.84	-0.23

JEDJIO	-4.03	0.67	-0.21
QAVWAM	-4.21	0.70	-0.20

Table S9 Calculated descriptors for 33 selected potential photocatalysts (unit: energy: eV, TOF: s^{-1})

Name	log(TOF)	$\Delta G_{\mathrm{H}}^{\mathrm{ads}}$	$\Delta G_{3-\mathrm{ref}}$	ΔG_{elec}	HOMO	LUMO	E_{S1}	f_{S1}	E_{ST}
VOCWAO	10.06	0.37	-1.16	-0.09	-6.64	-2.16	2.91	1.36	1.21
GEZCAP	9.60	0.12	-1.10	-0.06	-6.45	-1.85	2.97	1.24	1.30
XIBTOS	9.45	0.16	-1.08	-0.08	-6.99	-2.06	3.23	1.08	1.26
TIMCEA	9.43	0.37	-1.07	-0.43	-7.42	-1.69	3.09	0.00	0.86
BIFJAB	9.34	0.12	-1.06	-0.12	-6.74	-2.18	3.01	0.44	1.59
QEMYIR	8.91	0.10	-1.00	-0.18	-6.89	-2.08	3.14	1.26	1.43
MOMMUY	8.16	0.47	-0.89	-0.01	-6.47	-1.63	3.18	0.53	1.21
IRUJUC	7.70	0.40	-0.83	-0.04	-6.72	-2.00	3.28	1.04	1.16
EGUQED	7.53	0.27	-0.80	0.00	-6.82	-2.10	3.10	1.45	1.24
ZEVYIL	7.23	0.32	-0.76	-0.06	-6.69	-2.38	2.69	1.46	1.07
CIBTOZ	7.21	0.76	-0.76	-0.02	-6.81	-1.66	3.23	1.41	1.27
JOHFOC	7.19	0.42	-0.76	0.00	-6.80	-1.94	3.10	0.84	1.02
NAYQEK01	7.18	0.62	-0.75	-0.08	-6.54	-1.63	3.05	0.00	1.07
ZIGPIR	7.15	0.35	-0.75	-0.18	-6.60	-1.82	3.16	0.05	0.97
QAPNUT	6.99	0.32	-0.73	-0.11	-6.79	-2.14	2.99	1.19	0.95
FALNAI	-	0.49	-0.69	-0.10	-6.39	-1.89	2.86	0.89	1.27
FAPLIR	-	0.50	-0.94	-0.12	-6.97	-1.76	2.71	0.01	0.87
NUMRIZ	-	0.12	-0.93	-0.12	-6.67	-2.08	2.93	0.00	1.59
YAPGUR	-	0.49	-0.92	-0.20	-7.14	-1.75	2.72	0.00	0.83
BUVMAH	-	0.44	-0.89	-0.37	-7.42	-1.74	3.29	0.00	0.88
PUJCEE	-	0.70	-0.37	-0.31	-6.98	-1.90	3.22	0.05	1.22
YUFGOY	-	0.27	-0.94	-0.30	-6.98	-2.16	3.11	1.18	1.53
ABUHEN	-	0.65	-0.87	-0.04	-6.44	-1.95	3.10	1.17	1.49
FAXRII	-	0.48	-0.56	-0.15	-6.24	-2.12	2.40	0.00	1.20
SENJED	-	0.49	-0.51	-0.06	-6.71	-1.90	3.05	0.74	1.16

MELCEO	-	0.65	-0.44	-0.20	-6.81	-1.94	3.25	0.78	1.01
VUMTIJ	-	0.27	-0.44	-0.64	-7.50	-2.45	3.27	0.93	1.51
PUGCEC	-	0.45	-0.42	-0.39	-7.18	-2.38	3.09	0.52	1.30
BOHMET	-	0.64	-0.39	-0.05	-6.76	-1.96	3.10	0.95	1.19
BCNPPN10	-	0.22	-0.35	-0.13	-6.94	-2.58	2.45	0.00	0.98
NITVUK	-	1.16	-0.06	-0.02	-6.74	-1.96	3.15	1.67	1.25
ATUKUW	-	1.11	-0.01	-0.19	-6.81	-2.10	3.19	1.30	1.50
TAYGEG	-	0.46	-0.21	-0.05	-6.74	-2.39	2.83	2.24	0.98

Note: -: not found yet.

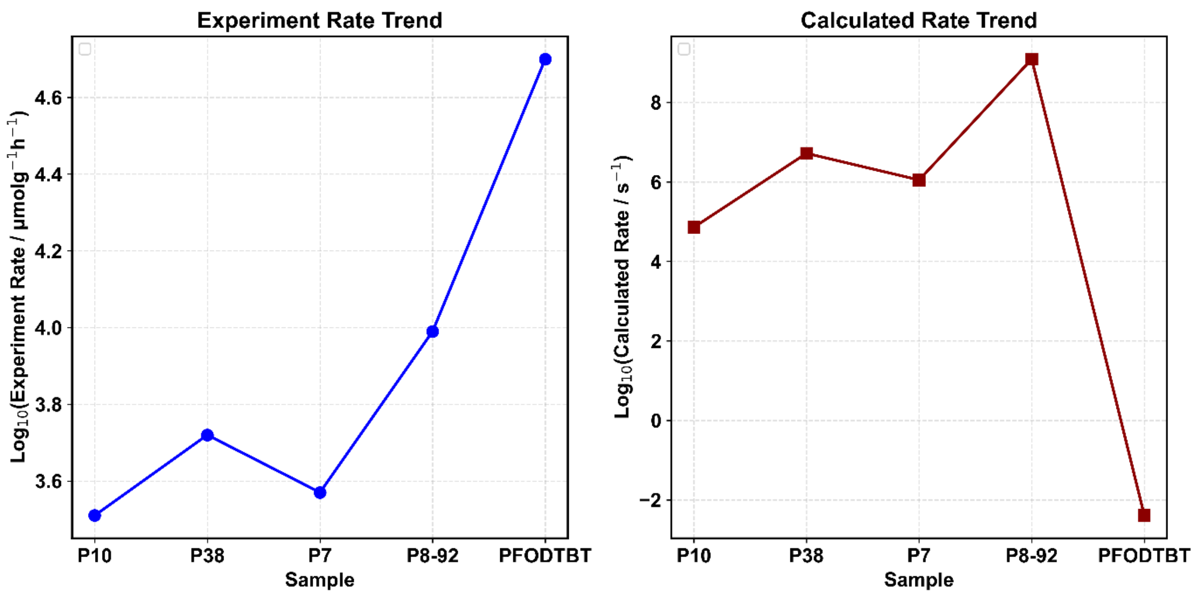

Figure S9. The comparison between **a**. The experimental rate trend and **b**. The calculated rate trend for P10, P38, P7, P8-92 and PFODTBT. The calculated rates were obtained from calculated monomers. The discrepancies could be attributed to the residue Pd (0.1% Pd (w/w)) for PFODTBT[[5]].

References

(1) Nørskov, J. K.; Studt, F.; Abild-Pedersen, F.; Bligaard, T. *Fundamental Concepts in Heterogeneous Catalysis*; Wiley, 2014.

(2) I. Chorkendorff, J. W. N. *Concepts of Modern Catalysis*; Wiley-VCH Verlag GmbH & Co. KGaA, 2003. DOI: 10.1002/3527602658.ch2.

(3) Wang, Z.; Hisatomi, T.; Li, R.; Sayama, K.; Liu, G.; Domen, K.; Li, C.; Wang, L. Efficiency Accreditation and Testing Protocols for Particulate Photocatalysts toward Solar Fuel Production. *Joule* **2021**, *5* (2), 344-359. DOI: 10.1016/j.joule.2021.01.001.

(4) Chase, M. *NIST-JANAF Thermochemical Tables, 4th Edition, J. Phys. Chem. Ref. Data, Monograph 9.*; 1998.

(5) Pati, P. B.; Damas, G.; Tian, L.; Fernandes, D. L. A.; Zhang, L.; Pehlivan, I. B.; Edvinsson, T.; Araujo, C. M.; Tian, H. N. An experimental and theoretical study of an efficient polymer nano-photocatalyst for hydrogen evolution. *Energ. Environ. Sci.* **2017**, *10* (6), 1372-1376. DOI: 10.1039/c7ee00751e.
